# Chemo-Phosphoproteomic Profiling with ATR Inhibitors Berzosertib and Gartisertib Uncovers New Biomarkers and DNA Damage Response Regulators

**DOI:** 10.1016/j.mcpro.2024.100802

**Published:** 2024-06-15

**Authors:** Rathan Jadav, Florian Weiland, Sylvie M. Noordermeer, Thomas Carroll, Yuandi Gao, Jianming Wang, Houjiang Zhou, Frederic Lamoliatte, Rachel Toth, Thomas Macartney, Fiona Brown, C. James Hastie, Constance Alabert, Haico van Attikum, Frank Zenke, Jean-Yves Masson, John Rouse

**Affiliations:** 1 MRC Protein Phosphorylation and Ubiquitylation Unit and School of Life Sciences, Wellcome Trust Biocentre, University of Dundee, Dundee, UK; 2 Department of Human Genetics, Leiden University Medical Center, Leiden, Netherlands; 3 Department of Genetics, Oncode Institute, Utrecht, The Netherlands; 4 CHU de Quebec Research Center, Oncology Division, Department of Molecular Biology, Medical Biochemistry and Pathology, Laval University Cancer Research Center, Quebec Cit, Quebec, Canada; 5 Division of Molecular, Cell and Developmental Biology, School of Life Sciences, Wellcome Trust Biocentre, University of Dundee, Dundee, UK; 6 EMD Serono, Research Unit Oncology, Billerica, Massachusetts, USA

**Keywords:** ATR, kinase, phosphoproteomic, SCAF1, RNAPII CTD, CHK1, homologous recombination, olaparib, berzosertib, gartisertib, biomarker

## Abstract

The ATR kinase protects cells against DNA damage and replication stress and represents a promising anti-cancer drug target. The ATR inhibitors (ATRi) berzosertib and gartisertib are both in clinical trials for the treatment of advanced solid tumors as monotherapy or in combination with genotoxic agents. We carried out quantitative phospho-proteomic screening for ATR biomarkers that are highly sensitive to berzosertib and gartisertib, using an optimized mass spectrometry pipeline. Screening identified a range of novel ATR-dependent phosphorylation events, which were grouped into three broad classes: (i) targets whose phosphorylation is highly sensitive to ATRi and which could be the next generation of ATR biomarkers; (ii) proteins with known genome maintenance roles not previously known to be regulated by ATR; (iii) novel targets whose cellular roles are unclear. Class iii targets represent candidate DNA damage response proteins and, with this in mind, proteins in this class were subjected to secondary screening for recruitment to DNA damage sites. We show that one of the proteins recruited, SCAF1, interacts with RNAPII in a phospho-dependent manner and recruitment requires PARP activity and interaction with RNAPII. We also show that SCAF1 deficiency partly rescues RAD51 loading in cells lacking the *BRCA1* tumor suppressor. Taken together these data reveal potential new ATR biomarkers and new genome maintenance factors.

## Highlights

• Phosphoproteomic screening using ATR inhibitors revealed new ATR substrates.
• Highly sensitive new targets could represent new pharmacodynamic ATR biomarkers.
• New ATR target SCAF1 interacts with phospho-CTD of RNA Pol II and is recruited to DNA damage sites.
• SCAF1 depletion partially restores PARP inhibitor resistance and HR in BRCA1-deficient cells.

## Graphical Abstract

**Figure:**
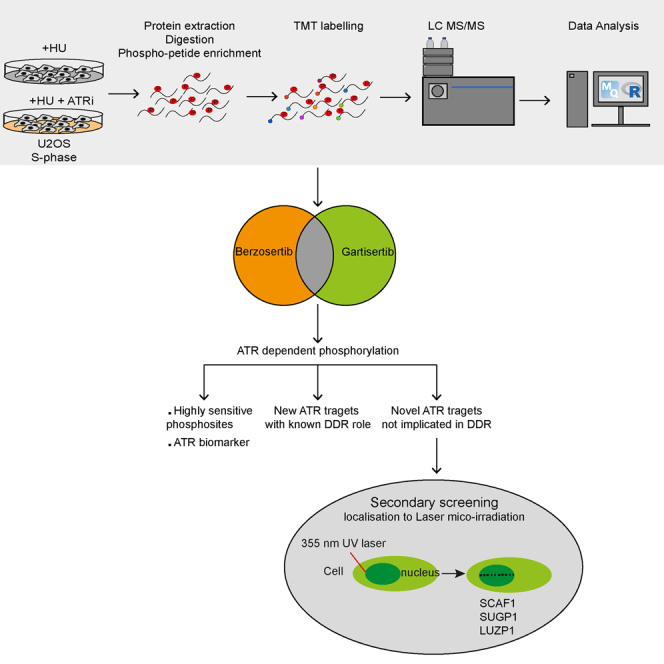

## In Brief

Phosphoproteomic screening using ATR inhibitors in clinical trials reveals new pharmacodynamic biomarkers and new DNA damage response proteins. Our study identified a role for SCAF1 in DNA damage repair, loss of which results in partial restoration of HR judged by RAD51 loading and PARP inhibitor resistance in BRCA1 deficient cells.

The ATR protein kinase plays a critically important role in the maintenance of genome stability (1, 2, 3). Through its targeting subunit ATRIP, ATR is recruited to sites in the genome where replisome progression is impeded (sites of replication stress; RS) (4, 5, 6). Stalled replication forks are prone to degradation and collapse, and a host of proteins including ATR is dedicated to protecting these structures so that replication can continue once the replisome-blocking impediment has been removed or bypassed (1, 7). Full loss of ATR causes cell lethality, probably because of catastrophic chromosome shattering during S-phase (6, 8), while hypomorphic mutations in ATR cause diseases such as Seckel syndrome (9). ATR belongs to the PI 3-kinase-related kinase (PIKKs) family which phosphorylates target proteins on Ser/Thr-Gln (S/T-Q) motifs (3, 10, 11, 12). A major target of ATR is CHK1, which is itself a kinase activated by ATR-mediated phosphorylation on several residues including Ser345 (13, 14). Together ATR and CHK1 play key roles in the stabilization of replication forks, and activation of cell cycle checkpoints to prevent entry to mitosis in the presence of excessive replication stress (15). In addition to its role as a key RS regulator, ATR is involved in inter-strand crosslink (ICL) repair, telomere control, DNA double-strand break (DSB) repair, and meiosis (16, 17, 18, 19).

Although it is an essential kinase, ATR has emerged over the years as a promising anti-cancer drug target (20, 21, 22, 23). Even though ATR activity is crucial in dealing with the low levels of RS in proliferating healthy cells, its activity becomes more important in tumor cells harboring activated oncogenes such as cyclin E (*CCNE1*), *MYC*, and *RAS* (24, 25, 26). This may reflect the elevated levels of RS in tumors resulting from the disruption of cell cycle regulation. In this light, several reports revealed that ATR inhibition is selectively toxic to tumors with high levels of DNA damage and RS (27, 28, 29, 30). Moreover, ATR inhibition was shown to be toxic in cancer cells harboring *ATM* mutations, a feature seen in many tumors (31, 32, 33), especially in combination with PARP inhibitors (32, 34). A range of ATR inhibitors has been developed in recent years including VE-821, BAY1895344, AZ20, AZD6738 (ceralasertib), and two inhibitors developed at Merck KGaA/EMD Serono - berzosertib (formerly known VE-822, VX-970, and M6620) and gartisertib (previously known as VX-803, M1774 and M4344). Given their potent anti-cancer activity in pre-clinical models (21, 35, 36, 37, 38, 39, 40, 41, 42, 43), berzosertib, gartisertib, BAY1895344, and ceralasertib have entered Phase I and II clinical trials. These trials are designed to test efficacy in combination with other drugs, but also as a monotherapy in a range of solid tumors, in particular those tumors with loss-of-function *ATM* mutations.

Pharmacodynamic biomarkers are important for evaluating target engagement in clinical trials. Phospho-CHK1 (pSer345) has worked as a biomarker to monitor ATR inhibition after berzosertib administration in combination with cisplatin to treat advanced solid tumors including PARP inhibitor-resistant *BRCA1*-mutated germline ovarian cancer and metastatic colorectal cancers with *ATM* or *ARID1A* mutations (44). However, pCHK1 was insufficiently sensitive to monitor the impact of berzosertib and gartisertib on ATR activity without genotoxic drug co-administration in clinical trials (45, 46, 47, 48, 49). Therefore, more sensitive ATR biomarkers are needed. In this light, a wide range of ATR targets have been identified by diverse phosphoproteomic screens using the ATR inhibitors VE-821 (50, 51, 52), AZ20 (46), AZD6738 (48), BAY1895344 (47) or using cell lines defective in ATR (49) or ATR activators (45). In principle, these datasets could be mined for new ATR biomarkers. Instead, we employed high-sensitivity, quantitative phosphoproteomic screening to look for proteins whose phosphorylation is highly sensitive to berzosertib and gartisertib. These analyses generated rich datasets, revealing a wide range of new ATR and CHK1 targets, and new players in the cellular response to DNA damage.

## Experimental Procedures

### Experimental Design and Statistical Analysis

For the global phosphoproteomics analysis, two screens were carried out. In the first screen, five biological replicates of each of two populations of S phase synchronized U-2 OS cells exposed to replication stress using hydroxyurea (HU) were used: one population was treated with ATR inhibitor gartisertib and another with DMSO vehicle as control. Thus, each biological replicate had two samples, giving a total of 10 samples. The second screen was the same except the ATR inhibitor berzosertib was used instead of gartisertib. From each of the 10 samples in each screen, 3.5 mg of protein extract was digested using Trypsin/LysC and phosphopeptides were enriched and labeled using TMT10plex. The pooled samples underwent prefractionation using high-pH RP-HPLC into 75 individual fractions, which were concatenated into 24 fractions. The fractions underwent analysis by LC-MS/MS in triplicate injections. TMT reporter intensities of phosphopeptides were quantified by MaxQuant and phosphopeptides measured several times within each of the 24 fractions were averaged. The resulting TMT reporter intensities were normalized using variance stabilizing normalization (VSN) and statistically tested using limma. Phosphopeptides showing lower abundance under inhibitor treatment and having an adjusted *p*-value ≤0.05 were deemed as potentially affected by ATR inhibitor treatment. A full description of the mass spectrometric methods for global phosphoproteomic screening, including sample preparation and data analysis is given in the Supplementary Materials.

### Reagents

All the reagents used in the current study including antibodies, siRNA sequences, cDNA clones, oligonucleotides, sgRNA sequences and peptides are listed in Supplemental Table S9.

### Cell Lines and Cell Culture

RPE1 hTERT *TP53*^*−/−*^ Cas9 and RPE1 hTERT *TP53*^*−/−*^ *BRCA1 KO* Cas9 cells were a kind gift of D. Durocher (Lunenfeld-Tanenbaum Research Institute, Mount Sinai Hospital). All the cell lines were grown at 37°C in a humidified incubator with 5% CO_2_. U-2 OS, U-2 OS Flp-In T-REx, RPE1 *hTERT* TP53^−/−^ Cas9 and RPE1 *hTERT TP53*^*−/−*^ *BRCA1 KO* Cas9 cells were cultured in high glucose Gibco Dulbecco's modified Eagle's medium (DMEM, Life Technologies) supplemented with 1 mM L-Glutamine, 10% (v/v) fetal bovine serum (FBS), 100 U/ml penicillin, and 100 μg/ml streptomycin. All cell lines were routinely tested and monitored for *mycoplasma* contamination.

### Cell Synchronization and Drug Treatment

U-2 OS cells were synchronized in the S phase by thymidine-nocodazole block. Briefly, cells were treated with 2 mM thymidine for 24 h, followed by release into fresh media for 3 h. Cells were then treated with 100 ng/ml nocodazole for a further 12 h. After treatment, cells that remained adherent were gently washed with PBS, released into fresh media for 11 h to enrich for S phase cells. Rounded and floating cells were collected, washed once in PBS and reseeded. S phase cells were either mock-treated or treated with 1 μM ATR inhibitors (listed in Supplemental Table S9) for 1 h, followed by replication stress induction with 1 mM hydroxyurea (HU) for 30 min. Following HU treatment cells were processed for global phosphoproteomic analysis (detailed in the Supplementary Materials & Methods section).

### Cell Cycle Analysis

Following thymidine-nocodazole block and release, cells were harvested by trypsinization at different times post-release. At each time point cells were fixed with ice-cold 70% ethanol overnight at −20 °C and stained with propidium iodide for 30 min at room temperature (PI; 50 μg/ml in PBS containing 5% FBS, 0.1 mg/ml RNase A). Cells were analyzed by flow cytometry (FACS canto, BD Biosciences) using DIVA software and data analysis to determine cell cycle phases was performed using FlowJo software.

### Cell Transfections

For transient expression of GFP-tagged proteins, 1 × 10^5^ U-2 OS or U-2 OS Flp-In T-REx cells were seeded in 35 mm glass bottom dishes (FD35-100, WPI); cells were transfected with 1 to 2 μg of pcDNA5 FRT/TO plasmids containing the gene of interest using GeneJuice transfection reagent (Cat#70967, Merck Millipore) according to manufacturer’s protocol. 8 h post-transfection, cells were induced with 1 μg/ml tetracycline hydrochloride for 24 h for protein expression. For siRNA-mediated protein knockdown, cells were transfected with 50 nM siRNA SMARTpools or individual siRNA using lipofectamine RNAi-Max transfection reagent (13778150, Invitrogen) according to the manufacturer’s protocol. Cells were analyzed or processed for downstream application after 48 to 72 h of transfection. siRNA sequences and source are provided in Supplemental Table S9.

### Stable Cell Lines Generation using Flp-In-T-REx System

Cells stably expressing GFP-tagged protein of interest were generated as previously described (Khanam *et al*, 2021). Briefly, U-2 OS Flp-In T-REx cells co-transfected with POG44 Flp-In recombinase expression vector and pcDNA5 FRT/TO - protein of interest in 9:1 ratio, using PEI Max transfection reagent (Cat#24765-100, Poly sciences). 48 h post-transfection, cells were selected and maintained using 100 μg/ml hygromycin and 10 μg/ml blasticidin in the medium. After 2 weeks, the surviving colonies were analyzed for target protein expression using tetracycline hydrochloride (Cat#T3383; Sigma-Aldrich). Stable cells expressing full-length and truncated versions of GFP-SCAF1 were generated in U-2 OS Flp-In T-REx cells stably expressing mCherry-XRCC1. Briefly, HEK-293FT packaging cells were co-transfected with pBabeD-Puro retroviral vector containing mcherry-XRCC1 along with GAG/Pol and VSVG constructs to generate retroviruses, using PEI Max transfection reagent. 48 h post-transfection medium containing virion particles was filtered, and target cells were transduced in the presence of 8 μg/ml polybrene for 24 h. Cells were selected using fresh media containing 1 μg/ml puromycin. Surviving cells were pooled, and single cells with low mCherry-XRCC1 expression were sorted using MA900 multi-application cell sorter (Sony Biotechnology).

### Immunoblotting

For the whole cell extracts, cell pellets were lysed on ice for 30 min in ice-cold RIPA buffer (10 mM Tris-HCl (pH 7.5), 150 mM NaCl, 0.5 mM EDTA, 0.1% SDS, 1% Triton X-100, 1% Sodium deoxycholate, 2.5 mM MgCl_2_) supplemented with protease inhibitor cocktail (cOmplete EDTA free protease inhibitor cocktail), phosphatase inhibitor cocktail-2 (Cat#P5726, Sigma-Aldrich) at 1% (v/v), universal nuclease (Cat#88700, Pierce Universal Nuclease) at a final concentration of 250 U/ml, microcystin-LR (Cat#33893, Sigma) at a final concentration of 10 ng/ml with intermittent mixing for every 10 min. Lysates were cleared by centrifugation at 17,000*g* for 10 min, supernatants were collected for protein estimation by BCA assay. 50 μg of total protein was mixed with a quarter of a volume of 4 × LDS sample buffer (Cat#NP0007, Invitrogen NuPAGE LDS Sample Buffer) and resolved on 4 to 12% Bis-Tris SDS PAGE gradient gels (NuPAGE, Thermo Fisher). Proteins were electrophoretically transferred onto 0.45 μm nitrocellulose membranes (Cat#10600002, Amersham Protran 0.45 um Nitrocellulose) at 200 mA constant current for 2 h on ice in Tris-Glycine transfer buffer with 20% (v/v) methanol, followed by blocking the membrane with 5% non-fat dry milk in TBS-Tween-20 (0.1% (v/v)) for 30 min at room temperature. The blots were probed with respective primary antibodies and incubated overnight at 4 °C (conditions for each primary antibody used in this study are listed in Table S9). The membrane was washed three times with excess of TBS-Tween-20 (0.1% (v/v)), probed with corresponding secondary antibody diluted in blocking buffer, and incubated for 1 h at room temperature. Blots were washed three times with TBS-Tween-20 (0.1% (v/v)) and once with 1× TBS prior to acquiring bands using the LI-COR Odyssey CLx Western Blot imaging system. Primary and secondary antibodies used for immunoblotting are listed in Supplemental Table S9.

**Fig. 1:**
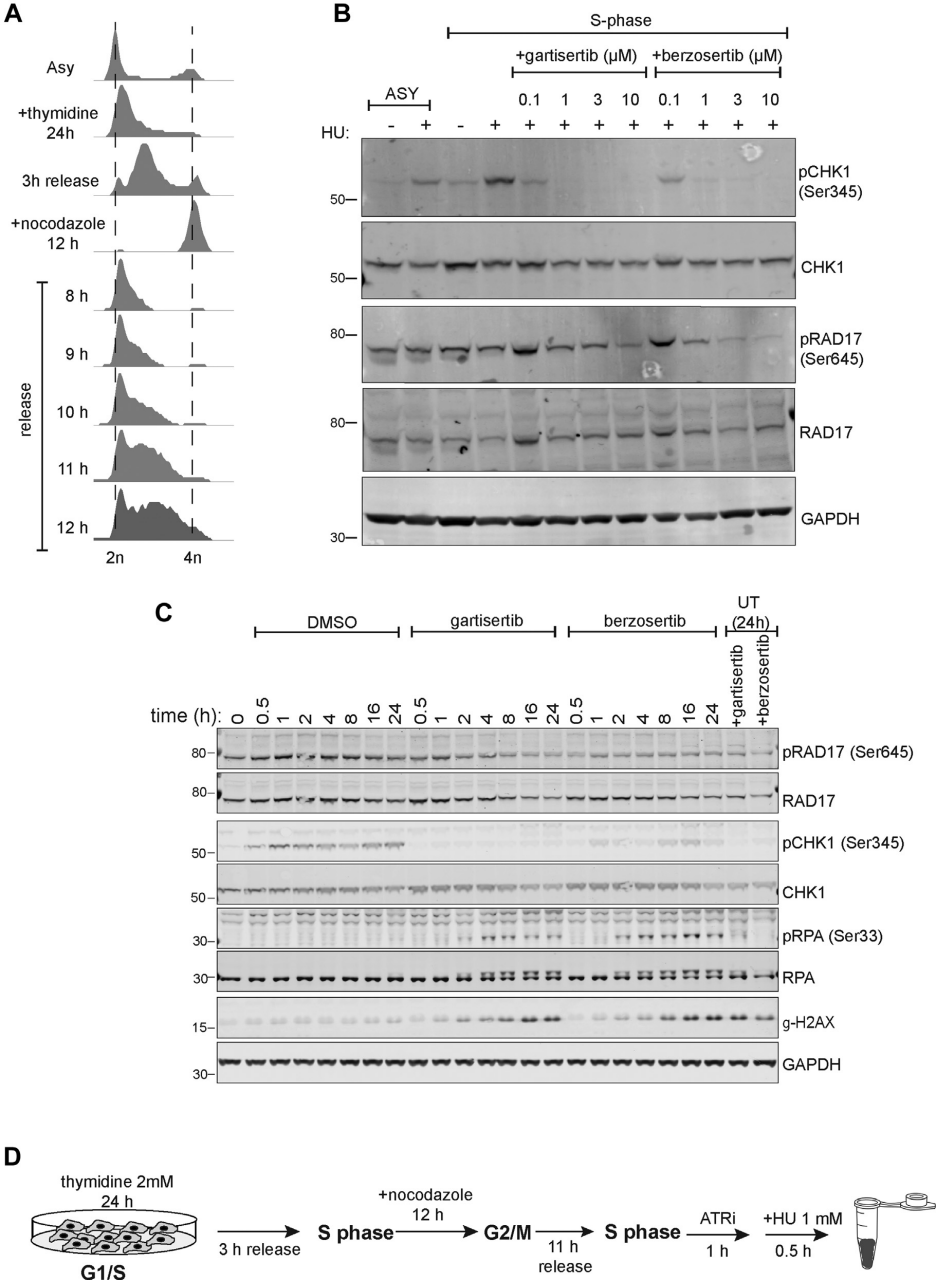
**Optimizing activation and inhibition of ATR**. *A*, U-2 OS cells were incubated with thymidine (2 mM) for 24 h and released for 3 h at which point nocodazole (100 ng/ml) was added for a further 12 h. Cells were released from nocodazole into fresh medium for the times indicated. Cells were fixed, stained with propidium iodide (PI) and analysed by FACS. *B*, U-2 OS cells synchronized in S-phase (11 h after release from nocodazole) were pre-incubated for 1 h with the indicated concentrations of berzosertib or gartisertib before addition of HU (1 mM) for 1 h. Cells were lysed, and extracts were subjected to Western blotting with the antibodies indicated. *C*, same as (*B*) except that S-phase cells were pre-incubated with berzosertib or gartisertib (1 μM for 1 h) before addition of HU (1 mM) for the times indicated. *D*, optimized cell treatment workflow.

**Fig. 2:**
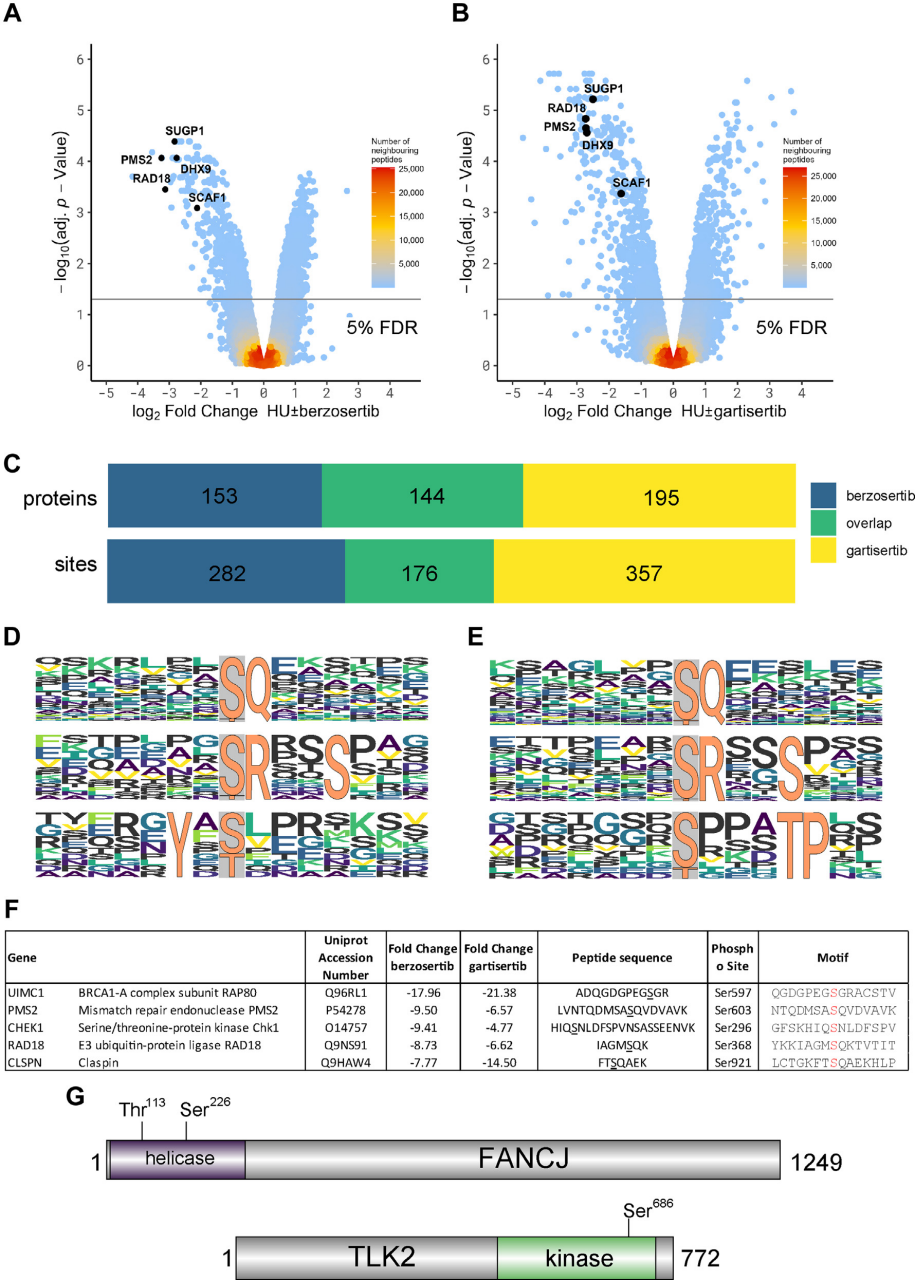
**Phosphoproteomic screening of phosphorylation sites sensitive to berzosertib or gartisertib**. *A* and *B*, Volcano plot showing phosphorylation sites affected by berzosertib (*A*) or gartisertib (*B*). The horizontal cut-off lines represent an adjusted *p*-value of 0.05. Phosphopeptides lower in abundance after inhibitor treatment are in the negative logFC region of the plots. The mass spectrometry proteomics raw data for this figure have been deposited to the ProteomeXchange Consortium (65) *via* the jPOSTrepo partner repository (64) with the dataset identifier PXD040469. Data analysis scripts and annotated spectra (66) are available *via* Zenodo under https://doi.org/10.5281/zenodo.10581948. *C*, overlap of proteins and phosphorylation sites affected by berzosertib or gartisertib. *D* and *E*, Phosphomotif analysis for berzosertib (*D*) and gartisertib (*E*). *F*, the five proteins whose phosphorylation is most strongly affected by berzosertib (“Class 1” hits). The phosphorylated residue is highlighted in red in the “Motif” column. Fold change refers to the difference between HU±ATRi. *G*, schematic diagram showing the location of the ATR-dependent phosphorylation sites in the DNA helicase FANCJ and the protein kinase TLK2.

For detection of ATR activation by immunoblotting using phospho-CHK1/phospho-Rad17 levels in Figures 1 and 2, cells either scraped or cell pellets were lysed directly in 1 × LDS sample buffer (Invitrogen NuPAGE LDS Sample Buffer, catalog number: NP0007) supplemented with 2% (v/v) 2-mercaptoethanol and samples were sonicated using a Bioruptor sonicator (Diagenode SA) at high amplitude for five 30 s on and off cycles. Samples were boiled at 95 °C for 5 min before proceeding for immunoblotting as described earlier.

### SCAF1 Gene Knockouts in RPE1 hTERT TP53^−/−^ Cas9 and BRCA1 KO Cas9 Cells

To knockout SCAF1 from cells, we identified two guide RNA sequences targeting exon 5 of the human SCAF1 gene. Single-guide RNA sequences were cloned into px459 GFP plasmid and cells were transfected with 2 μg of plasmid DNA (px459 GFP; sgRNA sequences in Supplemental Table S9) using PEI Max transfection reagent (Cat#24765-100, Poly sciences). 8 h post-transfection, media was replaced with fresh medium and cells allowed to grow for 48 h. Following 48 h of transfection, cells were harvested by trypsinization, processed for single cell sorting. GFP positive single cells were sorted in 96-well plates using an MA900 multi-application cell sorter (Sony Biotechnology). Single-cell clones were maintained in conditioned media with 20% FBS, at 37 °C humidified incubator with 5% CO_2_ until visible colonies formed. Single-cell clones of *BRCA1 KO* cells were grown under hypoxic condition with 3% CO_2_. Loss of protein was verified by immunoblotting and immunoprecipitation using SCAF1 polyclonal sheep antibody generated in house at DSTT (DA164, first bleed). Individual clones that showed no detectable SCAF1 protein were selected and genomic DNA around the exon five were amplified by PCR (Genotyping Primers Supplemental Table S9). PCR products were cloned using the StrataClone PCR cloning kit (Cat #240205, Agilent Technologies) and sequenced using T3 and T7 oligonucleotide to confirm the absence of wild type allele.

### GFP Pulldowns: SCAF1 Interaction with RNA Pol II

U-2 OS Flp-In T-REx cells stably expressing Tet-inducible GFP SCAF1 or GFP-SCAF1 (1187-1312) were induced overnight with 1 μg/ml tetracycline hydrochloride. Cells were treated with DMSO or with 10 μM Flavopiridol (Cat#S1230, Selleckchem) or 10 μM THZ1 (Cat#S7549, Selleckchem) for 4 h. Prior to harvest, cells were washed once with PBS and scraped on ice in cold lysis buffer (50 mM Tris-HCl (pH 7.4), 270 mM Sucrose, 150 mM NaCl, 1% (v/v) Triton X-100, 0.5% (v/v) NP-40, protease inhibitor cocktail, phosphatase inhibitor cocktail-2, 10 ng/ml microcystin-LR, benzonase (Novagen, 50 U/ml) and incubated 30 min on ice with intermittent mixing every 10 min by pipetting up and down. After 30 min, lysates were cleared by centrifugation at 17,000*g* for 10 min at 4 °C. The supernatant was collected, and protein concentration was estimated by the BCA assay. For anti-GFP immunoprecipitations, lysates were pre-cleared with Protein A/G Sepharose beads equilibrated with lysis buffer and incubated for 30 min at 4 °C on an end-over wheel. Pre-cleared lysates were used for immunoprecipitations using GFP-trap Sepharose beads (DSTT). Prior to immunoprecipitation, GFP-trap beads were washed twice in lysis buffer and incubated with precleared lysates (2 mg) for 90 min at 4 °C on the end-over wheel. Beads were washed three times for 3 min with ice-cold lysis buffer and a final wash with cold PBS. Immunoprecipitates were denatured by boiling samples in 2× LDS sample buffer supplemented with 2% (v/v) 2-mercaptoethanol at 95 °C for 5 min. Proteins were resolved by 4 to 12% SDS PAGE (NuPAGE) and electrophoretically transferred onto the nitrocellulose membrane. Immunoprecipitates and inputs were analyzed by immunoblotting using appropriate primary and corresponding secondary antibodies. Protein bands were acquired using LI-COR Odyssey CLx Western Blot imaging system.

### Peptide Pulldown Assays

Peptide binding assays were carried out using a biotinylated heptad repeat CTD peptide (Supplemental Table S9 for peptide sequence) either unphosphorylated or phosphorylated at S2 and S5 of the heptad (pCTD), and bacterial purified MBP or His_6_-tagged SCAF1 SRI domain (1187-end) protein. Briefly, 5 μg of non-phospho or phospho-CTD peptide (per condition) was conjugated to 10 μl (per condition) high-capacity streptavidin agarose beads (Cat#20357, Thermo Scientific) in peptide binding buffer (10 mM Tris-HCl pH 7.5, 150 mM NaCl, 270 mM sucrose, 0.1 mM EGTA, 0.1% (v/v) BME, 0.03% (v/v) Brij 35, protease inhibitor cocktail, phosphatase inhibitor cocktail-2) for 30 min at room temperature, and washed twice with an excess of peptide binding buffer. Beads were then incubated with 3 μg of purified MBP or 6His SCAF1 SRI (1187-end) protein in peptide binding buffer for 90 min at 4 °C on a thermomixer with continuous shaking at 1000 rpm. Beads were washed three times with excess of binding buffer followed by a final wash with ice cold PBS. Proteins were eluted by boiling beads in 2× LDS sample buffer at 95 °C for 5 min and resolved on 4 to 12% Bis-Tris SDS-PAGE gradient gels. Proteins were visualized by staining gel with InstantBlue Coomassie protein stain (Cat#ab119211; abcam). Lambda phosphatase treatment was carried out as indicated according to the manufacturer’s protocol. Peptide-bound beads were washed with 100 μl 1× lambda phosphatase buffer prior to incubating with lambda protein phosphatase (P0753; NEB).

### Peptide Pulldown: Endogenous SCAF1 Pulldown

Exponentially growing U-2 OS cells were harvested by trypsinization, washed once with ice-cold PBS, and nuclear fractionation was performed as described previously (53). After fractionation, nuclear pellets were lysed in nuclear lysis buffer (20 mM HEPES (pH 7.5), 300 mM NaCl, 1 mM DTT, protease inhibitor cocktail, phosphatase inhibitor cocktail-2, 0.5% NP-40 alternative, 250 U/ml Pierce Universal Nuclease, 10 ng/ml microcystin-LR, 10 μg/ml RNase A) for 30 min on ice. Nuclear lysates were sonicated using a BRANSON Digital Sonifier 450 at 35% amplitude for four 30 s on and off cycles. After sonication, the nuclear lysates were cleared by centrifugation at 20,000*g* for 10 min at 4 °C, and protein was estimated by BCA.

Nuclear lysates were subjected to peptide pulldown using biotinylated CTD peptide either unphosphorylated or phosphorylated at Ser2 and Ser5 (pCTD) or phosphorylated at Tyr1 of the heptad (pY1-CTD). Briefly, 5 μg of corresponding biotinylated CTD peptide was conjugated to 50 μl of Streptavidin Dynabeads (Cat#65601, Invitrogen) in binding buffer (PBS, 0.02% Tween-20) for 30 min at room temperature and washed twice with 1 ml of binding buffer. Beads were then incubated with 400 μg of nuclear lysates which were two times diluted with dilution buffer (20 mM HEPES (pH7.5), 0.2 mM EDTA, 1 mM DTT, Protease Inhibitor Cocktail, phosphatase inhibitor cocktail-2, 0.5% NP40 alternative, microcystin-LR) and incubated for 90 min at 4°C on a rotator. After incubation, beads were washed three times with wash buffer (20 mM HEPES (pH 7.5), 0.2 mM EDTA, 150 mM NaCl, 0.5% NP40 alternative) and a final wash with ice-cold PBS. Proteins were eluted with 2× LDS sample buffer. For Lambda phosphatase treatment, peptide bound beads were incubated with lambda phosphatase prior to pulldown.

### RAD51 Immunofluorescence (Fig. 6, *C* and *E*; JR lab)

For RAD51 immunofluorescence in Figure 6, *C* and *E*, RPE1 hTERT *P53*^*−/−*^ or RPE1 hTERT *P53*^*−/−*^ *BRCA1 KO* or *SCAF1 KO* cells were treated with respective siRNA where appropriate using RNAi Max transfection reagent according to manufacturer’s protocol. 24 h post-transfection, cells were trypsinized and counted, and 5000 cells/well were plated in Cell star clear flat bottom 96-well plates (Cat#655090, Greiner bio one) using 6 technical replicates per condition. After 36 h cells were treated with 10 Gy of IR and allowed to recover for different lengths of time (as indicated). At each time point cells were washed once with PBS, fixed with 3% paraformaldehyde in PBS (Santacruz) for 15 min at room temperature followed by two PBS washes and permeabilization with 0.2% Triton X-100 in PBS for 5 min at room temperature. Post permeabilization, cells were again washed twice with PBS followed by blocking with blocking buffer (DMEM (Gibco) +10% FBS) for 30 min at room temperature. Cells were co-stained with Rad51 (1:1000) and γH2AX (1:2000) primary antibodies in a blocking buffer for 2 h at room temperature followed by three washes with PBS. Cells were incubated with appropriate secondary antibodies diluted in blocking buffer with 1 μg/ml DAPI for 1 h at room temperature. Finally, cells were washed three times with PBS and were left in PBS until image acquisition. Images were acquired and analyzed with ScanR High Content Screening Microscopy (Olympus).

### RAD51 Immunofluorescence (Fig. 6*D*; SN lab)

Cells were transfected with siRNAs against SCAF1 using RNAiMax and grown on glass coverslips. 48 h post-transfection, cells were irradiated with 10 Gy and fixed 3 h post-IR. Simultaneously, RNA was isolated for qPCR to assess SCAF1 depletion. Cells were fixed for 20 min using 1% PFA, 0.5% Triton X-100 in PBS, followed by another 20 min using 1% (v/v) PFA, 0.3% (v/v) Triton X-100, 0.5% (v/v) methanol in PBS. Cells were blocked in PBS+ (5 g/L BSA, 1.5 g/L glycine in PBS) for 30 min at room temperature, followed by primary antibody incubation in PBS+ for 1.5 h at room temperature (1:15,000 Rb-anti-RAD51 (#70–001, BioAcademia) and 1:5000 M-anti-γH2AX (#05–636, Millipore)). Cells were incubated with fluorescently labeled secondary antibodies (1:1000 G-anti-Rb-AlexaFluor-488, G-anti-M-AlexaFluor-555, ThermoFisher Scientific) and DAPI in PBS+ for 1.5 h at room temperature before mounting with Aqua-Poly/Mount (Polysciences). Cells were imaged using a Zeiss Axio Imager 2 fluorescent microscope and cells with more than 5 RAD51 foci were manually counted.

### BrdU Foci

Cells were incubated with a medium containing 10 μM BrdU (Sigma) for 18 h, followed by no treatment or treated with 10 Gy irradiation and 3 h release. Cells were pre-extracted on ice for 8 min using two sequential extraction buffers. Pre-extraction buffer 1 (10 mM PIPES pH 7.0, 300 mM sucrose, 100 mM NaCl, 3 mM MgCl_2_, and 0.5% TritonX-100) and followed by pre-extraction buffer 2 (10 mM Tris-HCl pH 7.5, 10 mM NaCl, 3 mM MgCl_2_, 1% NP-40, and 0.5% sodium deoxycholate). Cells were washed three times with PBS followed by fixation with 4% paraformaldehyde (w/v) for 20 min on ice. After three PBS washes, cells were permeabilized in 0.5% TritonX-100 for 10 min and blocked in 3% BSA-PBS for 20 min on ice. Cells were then incubated with primary antibody against BrdU (1:1,000, Fisher Scientific) and PCNA (1:1,000, Novus) overnight at 4  °C, followed by three PBS washes and incubation with Alexa Fluor 488 goat anti-mouse (1:1,000, Thermo Fisher) and Alexa Fluor 568 goat anti-rabbit (1:1,000, Thermo Fisher) secondary antibody for 1 h at room temperature. Coverslips were mounted onto slides with ProLong Gold Antifade Mountant with DAPI (Invitrogen Life Technologies).

### pRPA (S4 and S8) Foci

The protocol was adapted from a previous report (54). Cells were treated with or without 10 Gy irradiation and 3 h release. Cells were then pre-extracted with buffer containing 25 mM HEPES pH 7.9, 300 mM sucrose, 50 mM NaCl, 1 mM EDTA, 3 mM MgCl_2_, and 0.5% TritonX-100 for 5 min on ice twice. Cells were fixed by 4% paraformaldehyde for 20 min at room temperature followed by another fixation with methanol for 5 min at −20  °C. After two PBS washes, permeabilization was carried out in 0.5% TritonX-100 for 15 min. After one wash with PBS supplemented with 0.1% Tween 20 (0.1% PBST), 2% BSA-PBS was used for blocking for 45 min. Cells were incubated with primary antibody against RPA2-phospho S4+S8 (1:500, Abcam) and PCNA (1:1,000, Novus) for 2 h at room temperature, followed by three washes in PBST and incubation with Alexa Fluor 488 goat anti-mouse (1:1,000, Thermo Fisher) as well as Alexa Fluor 568 goat anti-rabbit (1:1,000, Thermo Fisher) secondary antibody for 1 h at room temperature. Coverslips were mounted onto slides with ProLong Gold Antifade Mountant with DAPI (Invitrogen life technology).

### Protein Extraction and Immunoblotting (RPA Experiments in Supplemental Fig. S4*F*)

Cells were collected and lysed in lysis buffer containing 300 mM NaCl, 1% Triton X-100, 50 mM Tris-HCl pH8, 5 mM EDTA, and 1 mM DTT supplemented with protease inhibitors (1 mM PMSF, 3.4 μg/ml Aprotinin and 1 μg/ml Leupeptin) and phosphatase inhibitors (5 mM NaF and 1 mM Na_3_VO_4_) for 30 min on ice. Samples were sonicated using a Bioruptor sonicator (Diagenode) for 10 cycles (30 s ON/OFF at high power) and centrifugated for 20 min at 4 °C. Supernatants were collected and dosed by Bio-Rad Protein Assay Dye Reagent. Equal amounts of total protein were separated by SDS–PAGE and then transferred to nitrocellulose membrane (Bio-Rad) and immunoblotted with antibodies.

### Clonogenic Survival Assay

sgRNAs targeting SCAF1 (g1: CCACGGACAGCTTCCTCGCA; g3: CTCGGTGTCATGGCCTTCGA) were cloned into pLentiGuide-NLS-GFP as described before (Noordermeer *et al*., 2018). Viral supernatants were produced using HEK-293T cells upon jetPEI-mediated transfection (Polyplus, France) with pLentiGuide-NLS-GFP and third-generation packaging vectors. Viral supernatants were collected 48 h post-transfection. RPE1 hTERT *TP53*^−/−^ or *TP53*^−/−^ *BRCA1*^−/−^ cells virally expressing flag-Cas9 (Noordermeer *et al*., 2018) were transduced with the indicated sgRNAs (or empty vector as control) and transduced cells were selected using 10 (*TP53*^−/−^) or 15 (*TP53*^−/−^ *BRCA1*^−/−^) μg/ml puromycin for 6 days before seeding cells for clonogenic survival assays. DNA was collected to determine sgRNA targeting efficiency using genomic PCR of the targeting region and TIDE analysis (55). Targeting efficiencies were 58% for g1 and 70 to 78% for g3. Alternatively, cells were transfected with siRNAs against SCAF1 (Dharmacon, ON-TARGET plus SMARTpool; Horizon Discoveries) using RNAiMax (ThermoFisher Scientific). Clonogenic survival assays were seeded 48 h post-transfection. At the time of seeding, RNA was collected for qPCR using a commercially available TaqMan primer-probe for SCAF1 (ThermoFisher Scientific, Hs01553675_m1). 250 (*TP53*^−/−^) or 1500 (*TP53*^−/−^ *BRCA1*^−/−^) cells were seeded in 10 cm dishes for clonogenic survival in the presence or absence of 16 nM Olaparib and kept at 37 °C, 5% CO_2,_ and 3% O_2_. Medium with or without drugs was refreshed 7 days post-seeding. Colonies were stained after 14 days using crystal violet solution (0.4% (w/v) Crystal violet, 20% methanol) and manually counted.

### Endogenous SCAF1 Immunoprecipitation and Mass Spectrometry (IP/MS)

SCAF1 WT or KO cells were mock-treated or treated with 10 Gy of IR and allowed to recover for 1 h. Endogenous SCAF1 was immunoprecipitated using 10 mg lysate protein using anti-SCAF1 antibody (DA164, DSTT, University of Dundee). Cells were lysed in ice-cold lysis buffer (50 mM Tris-HCl (pH 7.4), 270 mM sucrose, 150 mM NaCl, 1% Triton (v/v) X-100, 0.5% (v/v) NP-40, protease inhibitor cocktail, phosphatase inhibitor cocktail-2, 10 ng/ml microcystin-LR, benzonase (Novagen, 50 U/ml) and incubated 30 min on ice with intermittent mixing by pipetting up and down every 10 min. After 30 min, lysates were cleared by centrifugation at 17,000*g* for 10 min at 4 °C. Supernatant was collected, and protein was estimated by the BCA assay. Samples were pre-cleared by incubating lysates with equilibrated Protein A/G beads for 30 min at 4 °C on an end-over wheel. SCAF1 was immunoprecipitated from pre-cleared lysates using sheep polyclonal SCAF1 antibody (first bleed, DA164, DSTT). Approximately 20 μg of anti-SCAF1 antibody (2 μg/mg of lysate) was conjugated to 50 μl of Protein A/G beads prior to performing immunoprecipitation. The conjugated antibody-bead complex was incubated with the lysates overnight at 4 °C for pull-downs. Immunoprecipitated complexes were washed three times with ice-cold lysis buffer and finally twice with cold PBS. Samples were boiled at 95 °C for 5 min in SDS lysis buffer (5% SDS in 100 mM triethylammonium bicarbonate pH 8.5, complete EDTA free protease inhibitor cocktail (Roche), phosphatase inhibitor cokatail-2, 1 μg/ml microcystin-LR). Eluates from the immunoprecipitates were further processed for S-trap assisted digestion using S-Trap micro spin column (Cat#C02-micro-40, Protifi) according to the manufacturer’s protocol followed by TMT labeling as described previously for the global phosphoproteomics screen (without phospho-peptide enrichment) (56). After TMT labeling, the quenched samples were mixed and fractionated with high pH reverse-phase C18 chromatography using the UltiMate 3000 high-pressure liquid chromatography system (Dionex) at a flow rate of 500 μl/min using two buffers: buffer A (5 mM ammonium formate, pH 10) and buffer B (80% ACN, 5 mM ammonium formate, pH 10). Briefly, the TMT-labeled samples were resuspended in 200 μl of buffer A (5 mM ammonium formate, pH10) and desalted then fractionated on a C18 reverse-phase column (4.6 × 250 mm, 3.5 μm, Waters) with a gradient as follows: 3% Buffer B for 19 min at 275 μl/min (desalting phase), ramping from 275 μl/min to 500 μl/min in 1 min, 3% to 12% buffer B in 1 min, 12% to 40% buffer B in 30 min, 40% B to 60% B in 5 min, 60% B to 95% B in 2 min, 95% for 3 min, ramping to 3% B in 1 min and then 3% for 9 min. A total of 96 fractions were collected and then concatenated into 24 fractions, which were further speed vacuum-dried prior to LC–MS/MS analysis. Peptides were resuspended in 5% formic acid in water and injected on an UltiMate 3000 RSLCnano System coupled to an Orbitrap Fusion Lumos Tribrid Mass Spectrometer (Thermo Scientific). Peptides were loaded on an Acclaim PepMap trap column (Thermo Scientific #164750) prior analysis on a PepMap RSLC C18 analytical column (Thermo Scientific #ES903) and eluted on a 120 min linear gradient from 3 to 35% Buffer B (Buffer A: 0.1% formic acid in water, Buffer B: 0.08% formic acid in 80:20 acetonitrile:water (v:v)). Eluted peptides were then analyzed by the mass spectrometer operating in Synchronous Precursor Selection mode using a cycle time of 3s. MS1 was acquired at a resolution of 120,000 with an AGC target of 100% and a maximum injection time of 50 ms. Peptides were then selected for MS2 fragmentation using CID with an isolation width of 0.7 Th, NCE of 35%, AGC of 100%, and maximum injection time of 50 ms using the “rapid” scan rate. Up to 10 fragments were then selected for MS3 fragmentation using HCD with an isolation width of 3 Th, NCE of 65%, AGC of 200%, and maximum injection time of 105 ms, and spectra were acquired at a resolution of 50,000. Dynamic exclusion was set to 60 s with a tolerance of ± 10 ppm. Mass spectrometry raw data was searched using MaxQuant (version 2.1.3.0) (57) against a *homo sapiens* FASTA (42,390 entries, downloaded 18th August 2022, inclusive protein isoforms) from Uniprot (www.uniprot.org). Additionally, to the default MaxQuant search parameters (digestion enzyme: Trypsin/P, maximum of 2 missed cleavages), as variable modification oxidation of methionine was set, and as fixed modification, carbamylation of cysteine was selected. Deamidation of N and Q was additionally set as variable modification. As per default settings, mass tolerance for the precursor ions for the first search was limited to 20 ppm. The main search tolerance of 4.5 ppm was set, while the MS/MS tolerance was 20 ppm. The peptide false discovery rate (FDR) and protein FDR were set to 5%. Data was analyzed using R (version 4.1.1) (58) with in-house developed scripts based on previous versions published (56). In brief, intensities of peptides repeatedly measured within a single fraction were averaged. Data was then transformed and calibrated using VSN (59, 60). The median peptide intensities belonging to each respective protein were taken as heuristics for total protein intensity, and data were statistically tested using limma (61, 62). Proteins underwent volcano plot analysis and proteins clustering within a group distinct from the bulk of data (63) were regarded as statistically significant (adjusted *p*-value <0.08). Mass spectrometry raw data, MaxQuant search parameters and output file, and FASTA file have been deposited at jPOSTrepo (64) and can be downloaded *via* ProteomeXchange (65) (PXD041201). All data analysis scripts and annotated spectra (generated using PDV version 1.8.2 (66)) can be downloaded from Zenodo (67) *via* https://doi.org/10.5281/zenodo.10581731.

### Extracted Ion Chromatography (XIC) Analysis

U-2 OS cells were seeded at 25% confluency in 100 mm plates. 24 h post-plating, cells were transiently transfected with plasmid DNA containing GFP tagged protein of interest using PEI Max transfection reagent (24765; Polysciences). Briefly, 3 μg of plasmid DNA and 9 μg of PEI (1:3 ratio of DNA:PEI) were diluted in 1 ml of Opti-MEM reduced serum media, pulse vortexed for 15 s and the mixture was incubated at room temperature for 15 min. The transfection mixture was then added dropwise to the target cells, 8 h post-transfection fresh media was replaced, and cells were incubated further for 24 h. After 24 h, cells were either mock-treated or treated with 1 μM gartisertib or 0.5 μM CHK1 inhibitor PF477736 for 1 h followed by hydroxyurea treatment at a final concentration of 1 mM for 30 min. Cells were then harvested by trypsinization and washed once with ice-cold PBS. Cell pellets were lysed in 300 μl of RIPA buffer (10 mM Tris-HCl (pH 7.5), 150 mM NaCl, 0.5 mM EDTA, 0.1% (v/v) SDS, 1% (v/v) Triton X-100, 1% (v/v) sodium deoxycholate, 2.5 mM MgCl_2_, Protease inhibitor cocktail, phosphatase inhibitor cocktail-2, 10 ng/ml microcystin-LR) on ice for 30 min and then diluted with 450 μl of dilution buffer (10 mM Tris-HCl (pH 7.5), 150 mM NaCl, 0.5 mM EDTA, Protease inhibitor cocktail, phosphatase inhibitor cocktail-2), incubated at 4 °C on end-over wheel for further 10 min. Lysates were then clarified by centrifugation at 13,300 rpm for 10 min at 4 °C. An aliquot of supernatant was saved for immunoblot analysis.

For extracted ion chromatography (XIC) analysis, 25 μl of equilibrated GFP-trap Sepharose beads (DSTT, University of Dundee) were incubated with lysates for 90 min at 4 °C. Precipitates were washed three times with 1 ml ice-cold washing buffer (4 parts of RIPA buffer mixed with 6 parts of dilution buffer). Samples were denatured in 2× LDS sample buffer supplemented with 2% (v/v) 2-mercaptoethanol at 95 °C for 5 min. The experiment was carried out in triplicates using lysates from independent replicates per condition. Denatured samples were resolved in 4 to 12% Bis-Tris SDS-PAGE gradient gels and stained with InstantBlue Coomassie protein stain (Cat#ab119211, Abcam). Protein bands were excised from gel, cut into approximately 1 mm^2^ pieces, and destined using 25 mM AmBiC, 30% (v/v) acetonitrile solution. Proteins were digested with trypsin/LysC protease (Cat#A40009; Thermo Scientific) and labeled with TMT 10plex as described previously (56). The data analysis protocol is described in detail in Supplementary Materials and Methods. The mass spectrometry raw data for LUZP1 and SUGP1 was uploaded to ProteomeXchange *via* jPOSTrepo can be downloaded from with the identifiers PXD040476, while DHX9 data was uploaded *via* PRIDE (68) (PXD041250), and jPOSTrepo (PXD050953 and PXD050954). The data analysis scripts, and annotated spectra (66) are available *via* Zenodo (https://doi.org/10.5281/zenodo.10581706 (LUZP1, SUGP1) and https://doi.org/10.5281/zenodo.10882997 (DHX9).

### Laser Micro-Irradiation

Recruitment of protein to the site of DNA damage was monitored by laser micro-irradiation as described previously (63) with the following changes. Around 1 x 10^5^ U-2 OS Flp-In T-REx cells were seeded in 3.5 cm glass bottom dishes (FD35–100) and transiently transfected with the plasmid DNA containing GFP-tagged protein of interest (See Supplemental Table S9 for plasmid constructs) using GeneJuice transfection reagent (70967, Merck) according to manufacturer’s protocol. After 24 h, the media was changed to complete DMEM containing 10 μM bromodeoxyuridine (BrdU) for another 24 h. Shortly before irradiation, media was replenished with warm phenol-red free media (31053; Thermo Fisher). Cells were placed in a 37 °C chamber incubator supplemented with 5% CO_2_ mounted on a Leica TCS SP8X microscope system (Leica Microsystems).

For SCAF1 recruitment, cells stably expressing GFP-SCAF1 were seeded in 3.5-cm glass bottom dish in the presence of 10 μM BrdU and 1 μg/ml tetracycline hydrochloride for 24 h. Prior to irradiation cells were either mock-treated or treated with flavopiridol, THZ1, DRB, olaparib, or PDD00017273 (PARGi) as indicated, and cells were placed on a chamber incubator at 37 °C with 5% CO_2_ attached to an Axio Observer Z1 spinning disc confocal microscope (Zeiss). Image acquisition and image analysis were performed as described in (63).

## Results

### Optimizing ATR Activation and Inhibition

We set out to establish optimal conditions for activating and inhibiting ATR in U-2 OS osteosarcoma cells. We first synchronized cells in S-phase, as ATR is activated at this phase of the cell cycle. To this end, cells were released from a 24 h thymidine block into nocodazole for 12 h; approximately 11 h after release from the nocodazole-induced G_2_ arrest, the majority of cells were in S-phase (Fig. 1*A*). As shown in Fig. 1*B*, addition of hydroxyurea to S-phase synchronized cells caused a higher level of ATR activation compared with asynchronous cells, judged by CHK1 Ser345 phosphorylation (compare lane 4 with lane 2). To avoid inducing DSB (which would activate ATM and DNA-PK) we sought to use the lowest HU exposure time necessary to fully activate ATR, and the lowest dose of ATRi needed for full inhibition. As shown in Fig. 1*C*, a 30 min HU treatment was sufficient to activate ATR, judged by CHK1 (pSer345), and pre-incubation of cells with 1 μM berzosertib or gartisertib for 1 h prior to HU was sufficient to fully block phosphorylation of CHK1 pSer345 (Fig. 1*B*). Under these conditions, no increase in phosphorylation of H2AX, a marker associated with DSB formation, or phospho-RPA, a marker of DNA end resection, was detected (Fig. 1*C*). Based on these data, we settled on the cell treatment workflow shown in Fig. 1*D* for phosphoproteomic screening.

### Phosphoproteomic Screening for Phosphorylation Events Inhibited by Berzosertib and Gartisertib

We first carried out a quantitative phosphoproteomic screen, comparing cells exposed to [HU+DMSO] and [HU+berzosertib] according to the pipeline shown in Supplemental Fig. S1*A*. Five biological replicates of each of the 2 cell populations were lysed (Supplemental Fig. S1*B*, left panels), and Cys residues were reduced and alkylated. After trypsinization of cell extracts, phosphopeptides were enriched by titanium dioxide chromatography. The 10 samples were then isotopically labeled with tandem mass tags (TMT), allowing multiplexed and quantitative analysis of all 10 samples which were combined and analyzed in parallel (68). Applying a false discovery rate (FDR) threshold of 5% identified 21,178 unique phosphopeptides of which 17,128 had at least one phosphorylation site with a localization probability of ≥75% (14,251 unique sites); this yielded 9367 unique phosphorylation sites with a 1% false localization rate, as called by MaxQuant (Supplemental Tables S1 and S2). Normalization and intensity distribution in the TMT channels were checked and deemed satisfactory (Supplemental Fig. S1, *C* and *E*). To define berzosertib-sensitive phosphorylation events, mass spectrometric data were visualized in a volcano plot, which revealed 553 phosphopeptides that were lower in abundance after exposure of cells to berzosertib (Fig. 2*A* and Supplemental Table S2). These 553 phosphopeptides corresponded to 463 unique sequences because phosphorylation of different residues in a single unique sequence can give rise to more than one phosphopeptide from that sequence. All the phosphopeptides within this group had an adjusted *p*-value of <0.05 (5% FDR).

A second phosphoproteomic screen was carried out with cells exposed to [HU+DMSO] or [HU+gartisertib] according to the pipeline shown in Supplemental Fig. S1*A*, again with five biological replicates per condition (Supplemental Fig. S1*B*, right panels). Applying an FDR of 5% identified 22,853 unique peptides of which 17,743 had at least one phosphorylation site with a localization probability of ≥75% (14,456 unique sites); this yielded 8924 unique phosphorylation sites with a 1% false localization rate (Supplemental Tables S1 and S3). Normalization and intensity distribution in the TMT channels were checked and deemed satisfactory (Supplemental Fig. S1, *D* and *F*). To define gartisertib-sensitive phosphorylation events, mass spectrometric data were visualized in a volcano plot, which revealed 657 phosphopeptides (559 unique sequences) that were lower in abundance after exposure of cells to gartisertib (Fig. 2*B* and Supplemental Table S3); all the phosphopeptides within this cluster had an adjusted *p*-value of <0.05 (5% FDR). Comparison of the phosphorylation sites inhibited by berzosertib or gartisertib revealed an overlap of around 45% corresponding to 176 sites in 144 target proteins (Fig. 2*C*). The full list of overlapping hits is given in Supplemental Table S4, and the overlapping hits with a fold change of greater than 4 are listed in Table 1. Given these sites were inhibited by the two different ATRi we used, we regard them as *bona fide* ATR-dependent phosphorylation events.

**Table 1:** ATRi phosphoproteomic dataset: top hits

List of the phosphorylation sites inhibited by both berzosertib and gartisertib, with a fold change greater than 4, ranked according to fold-change with berzosertib. Phosphorylation sites are highlighted in red in the “Motif” column.

Analysis of the amino acid sequences surrounding the phosphorylation sites inhibited by berzosertib and gartisertib revealed strong enrichment of two different phospho-motifs common to both inhibitors: the pS/pT-Q motif typical of ATR and other PI-(3) kinase-like kinases, and a pSRXXS motif where the first serine is the phosphorylated residue (Fig. 2, *D* and *E*). This latter motif is somewhat reminiscent of the overrepresentation of SR motifs among ATR targets in IR-treated HL-60 cells (50). Phosphorylation of this motif is presumably targeted by an ATR-activated kinase other than CHK1 which phosphorylates S/T residues in an RXXS motif *in vitro* and in cell extracts; CHK1 should not phosphorylate the first Ser in the pSRXXS motif (69, 70, 71). Intriguingly, each ATRi inhibited phosphorylation of Ser/Thr residues in a third phospho-motif that was unique to each inhibitor: YX[pS/pT] for berzosertib and pSXXXTP for gartisertib (Fig. 2, *D* and *E*). It is possible these motifs reflect off-target effects uniquely associated with each inhibitor. As expected, gene ontology (GO) analysis showed a striking enrichment of the terms DNA repair, DNA replication, and double-strand break repair (NHEJ and HR) for both ATRi (Supplemental Fig. S2*A*). The interaction network (72) of the proteins with phosphorylation sites lower in abundance after the respective ATR inhibitor treatment, revealed kinases, E3 ligases, deubiquitinases and previously known target proteins of ATR, ATM, CHK1 and CHK2 (Supplemental Figs. S5 and S6).

We were interested to see that a range of phosphorylation sites increased in cells treated with the two different ATRi (Supplemental Fig. S7, Supplemental Tables S2, and S3). We found 583 phosphorylation sites (379 proteins) that were higher in abundance after berzosertib treatment, while 683 phosphorylation sites (475 proteins) were higher in abundance after gartisertib, with 158 sites (175 proteins) common to both inhibitors (Supplemental Fig. S7*A*, Supplemental Tables S7, and S8). When applying a 1% FLR, 355 phosphorylation sites remained in the gartisertib experimental series, and 12 upregulated sites (3.4%) conformed to the PIKK consensus pS/pT-Q motif, and this was 1.9% sites after berzosertib (11 out of 592). Analysis of the amino acids surrounding the phosphorylation sites upregulated after either berzosertib or gartisertib identified three distinct motifs: SXXXpS/pTP; pS/pTXXK and pS/pTPXXK (Supplemental Fig. S7*B*) typically phosphorylated by cyclin-dependent kinases (73, 74). In this light, gene ontology terms common to phosphosites upregulated by gartisertib and berzosertib included cell division and mitotic cell cycle (Supplemental Fig. S7*C*). These data could be explained by ATR inhibition reversing CDK inhibition that is normally triggered by replication stress (HU). Intriguingly, interaction network analysis (Supplemental Fig. S8) also revealed the presence of four kinases (PBK, PRKDC [DNA-PK], PDPK1, and PKN1) whose phosphorylation is increased after ATRi, but no E3 ligases, deubiquitinases or previously known targets of ATR, ATM, CHK1 and CHK2 (Supplemental Tables S2 and S3).

### ATRi-Sensitive Targets Fall into Three Classes

We focussed our attention on phosphorylation sites that were downregulated in response to both ATRi, and we grouped the hits into three classes. The first class is those showing the highest degree of sensitivity to ATRi (>four-fold change ± ATRi), and these sites have good potential as new ATR biomarkers (Table 1). The top 5 berzosertib-sensitive sites, that are also sensitive to gartisertib, are shown in Figure 2*F*. The most sensitive phosphosite is pSer597 of RAP80, a subunit of the BRCA1-A complex (75), which displayed a fold change in phosphorylation of 21 and 18 with berzosertib and gartisertib respectively. This was not previously known as an ATR-dependent phospho-site. PMS2 pSer603 and RAD18 pSer368, which are also among the top 5 phosphorylation sites with the highest sensitivity to ATRi, both lie in classical SQ motifs and are therefore likely to be direct ATR targets. Also featured in the top 5 was CHK1 pSer296, a known DNA damage-induced autophosphorylation site (76).

The second class of ATRi-sensitive phosphorylation sites (fold change >1.5 ± ATRi) are those found in proteins already implicated in cellular DNA damage responses, but not known previously as ATR-dependent phosphorylation sites (Supplemental Table S5). Examples of proteins in this category include MCM9 which interacts with MCM8 to form a heterohexamer (paralogous to the MCM2-7 replicative helicase) involved in post-synaptic DNA synthesis during homologous recombination (HR) (77); the SLX4 scaffold protein which tethers and coordinates three structure-selective DNA repair nucleases (SLX1, XPF–ERCC and MUS81–EME1) (78); RAP1 (TERF2IP), which interacts with the shelterin component TRF2 to regulate telomere length and protection (79). Of particular interest in this category are novel ATR-catalyzed phosphorylation sites within functionally annotated catalytic domains. For example, FANCJ is a BRCA1-associated, DNA-dependent ATPase and helicase involved in HR and ICL repair (Fig. 2*G* and Supplemental Table S5) (80, 81). In this study we found that FANCJ is phosphorylated on two residues in the helicase catalytic domain – Thr113 and Ser226, both of which conform to the classical S/T-Q ATR consensus motif. The kinase TLK2, implicated in chromatin assembly, replication fork integrity, and recovery from DNA damage-induced G_2_ arrest is phosphorylated on Ser686 (Fig. 2*G* and Supplemental Table S5) (82, 83). This is a highly conserved residue within the kinase catalytic domain, raising the possibility that ATR regulates TLK2 kinase activity.

**Fig. 3:**
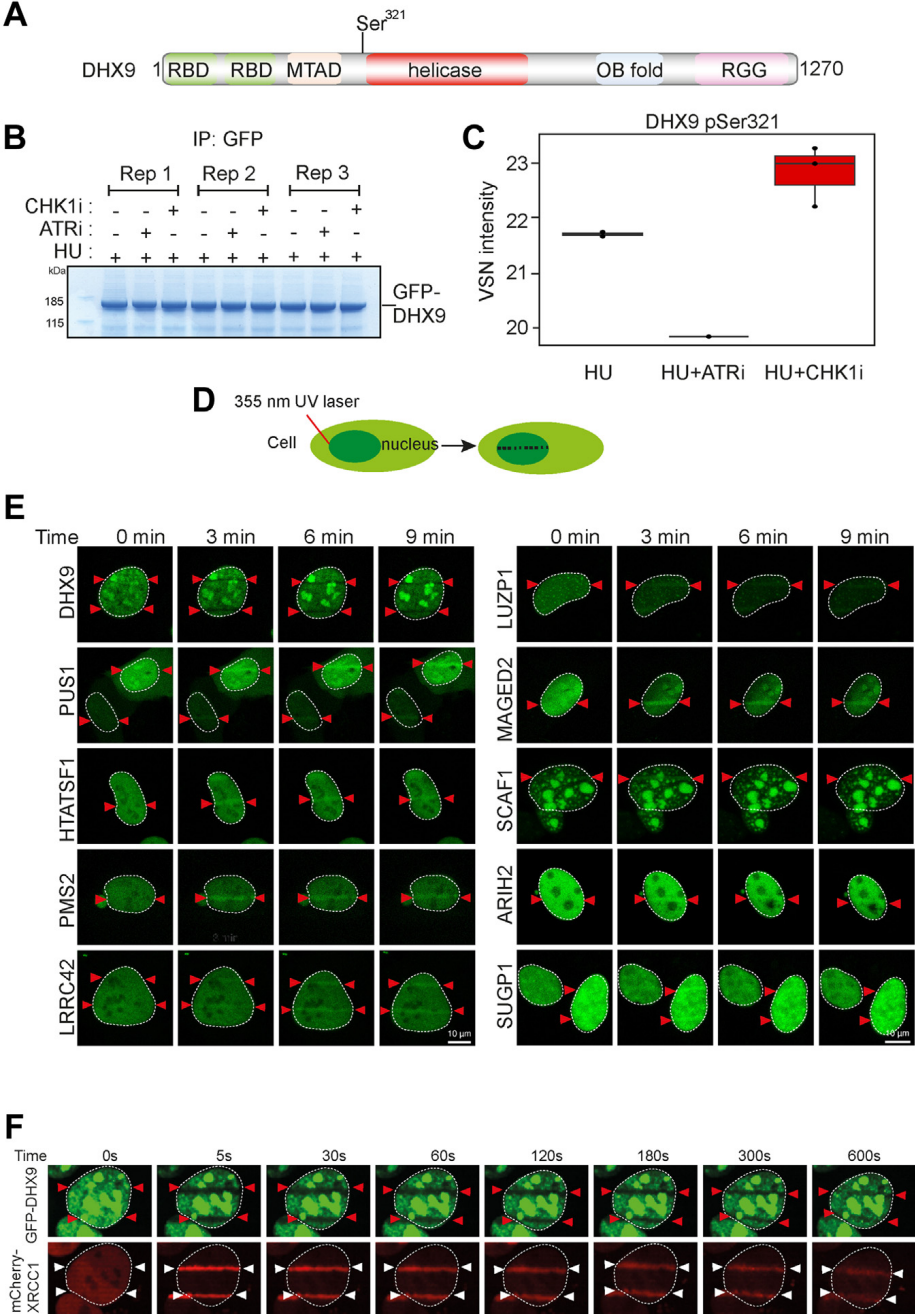
**Screening novel ATR targets for recruitment to DNA damage sites**. *A*, schematic diagram showing the domain organization of DHX9. RBD, RNA binding domain; MTAD, minimal transcriptional activation domain; RGG, RGG-rich domain; OB fold, oligonucleotide/oligosaccharide binding fold. *B*, U-2 OS cells were transfected with GFP-tagged DHX9 and after 24 h cells were lysed, and cell extracts were subjected to immunoprecipitation with anti-GFP-agarose beads. Precipitates were subjected to SDS-PAGE and staining with Coomassie Brilliant Blue; the bands corresponding to the GFP-DHX9 were excised and processed for mass spectrometric detection of relevant phospho-peptides. Three independent co-transfection experiments were done for every condition (Rep = biological replicate). *C*, label-free quantification was used to generate a boxplot showing VSN transformed intensity of phospho-peptides containing to DHX9 pSer321. Mass spectrometry raw data was uploaded to ProteomeXchange *via* the PRIDE partner repository (126) and can be downloaded *via* the identifier PXD041250. Data analysis scripts and annotated spectra (66) can be accessed *via* Zenodo under https://doi.org/10.5281/zenodo.10882997. *D*, schematic diagram showing micro-irradiation of BrdU-sensitized cells to induce DNA damage along a track in the nucleus. *E*, BrdU–sensitized U-2 OS cells transiently expressing GFP-tagged forms of the proteins indicated were line micro-irradiated and imaged after 2 min. *F*, BrdU-sensitized U-2 OS cells stably expressing mCherry-XRCC1 and expressing GFP-DHX9 in a tetracycline-inducible manner were subjected to laser micro-irradiation and live imaged at the times indicated.

We also noticed that the DHX9 helicase is phosphorylated in an ATR-dependent manner on Ser321 which lies in a classical SQ consensus motif (Table 1 and Supplemental Table S5). DHX9 is a poorly understood helicase capable of unwinding DNA, RNA, and hybrid nucleic acids *in vitro*, with pleiotropic roles in the maintenance of genome stability (84). Recently, DHX9 was shown to facilitate R-loop formation and to stimulate BRCA1-dependent DNA end resection and HR (85, 86). Ser321 lies close to the start of the helicase domain suggesting it may influence DHX9 activity and/or function (Fig. 3*A*), and we next sought to validate ATR-dependent phosphorylation of this site on DHX9 expressed in U-2 OS cells. XIC analysis of tryptic phosphopeptides isolated from GFP-tagged DHX9 (pSer321) confirmed that the HU-induced phosphorylation of this site is reduced by preincubating cells with berzosertib but not with the CHK1 inhibitor PF477736 (87) (Fig. 3, *B* and *C* and Supplemental Fig. S2, *B* and *C*). We obtained similar data with DHX9 isolated from HEK-293 cells or HeLa cells (Supplemental Fig. S2, *E* and *F*), and therefore, DHX9 is a target of ATR, consistent with a recent report (88).

The third class of ATR-dependent phosphorylation sites (fold change >1.5 ± ATRi) are found in proteins that were not previously linked to cellular responses to DNA damage or replication stress (Supplemental Table S6). Several proteins in this class are of unknown function, and we focussed on some of these.

### Secondary Screening: Recruitment to DNA Damage Sites

All of the proteins in the third class of ATR targets mentioned above are potentially new DDR proteins. Re-localization to DNA damage sites is a universal feature of proteins involved in DDR, and we next tested a range of class iii proteins for this behavior. Cells prelabeled with BrdU expressing GFP-tagged versions of each protein were subjected to micro-irradiation to induce DNA damage along a track in the nucleus with a 355 nm laser (Fig. 3*D*). Some of the proteins tested showed robust recruitment to DNA damage sites (Fig. 3*E* and Supplemental Fig. S2*D*). For example, pseudouridine synthase PUS1, and the E3 ubiquitin ligase ARIH2 are recruited rapidly and in a sustained manner to DNA damage sites and may therefore play previously unanticipated roles in the DDR (Fig. 3*E* and Supplemental Fig. S2*D*). DHX9 was the only protein we found to be excluded from DNA damage sites (Fig. 3*F*); the underlying mechanism is not yet known but exclusion is not prevented by berzosertib or gartisertib (data not shown).

**Fig. 4:**
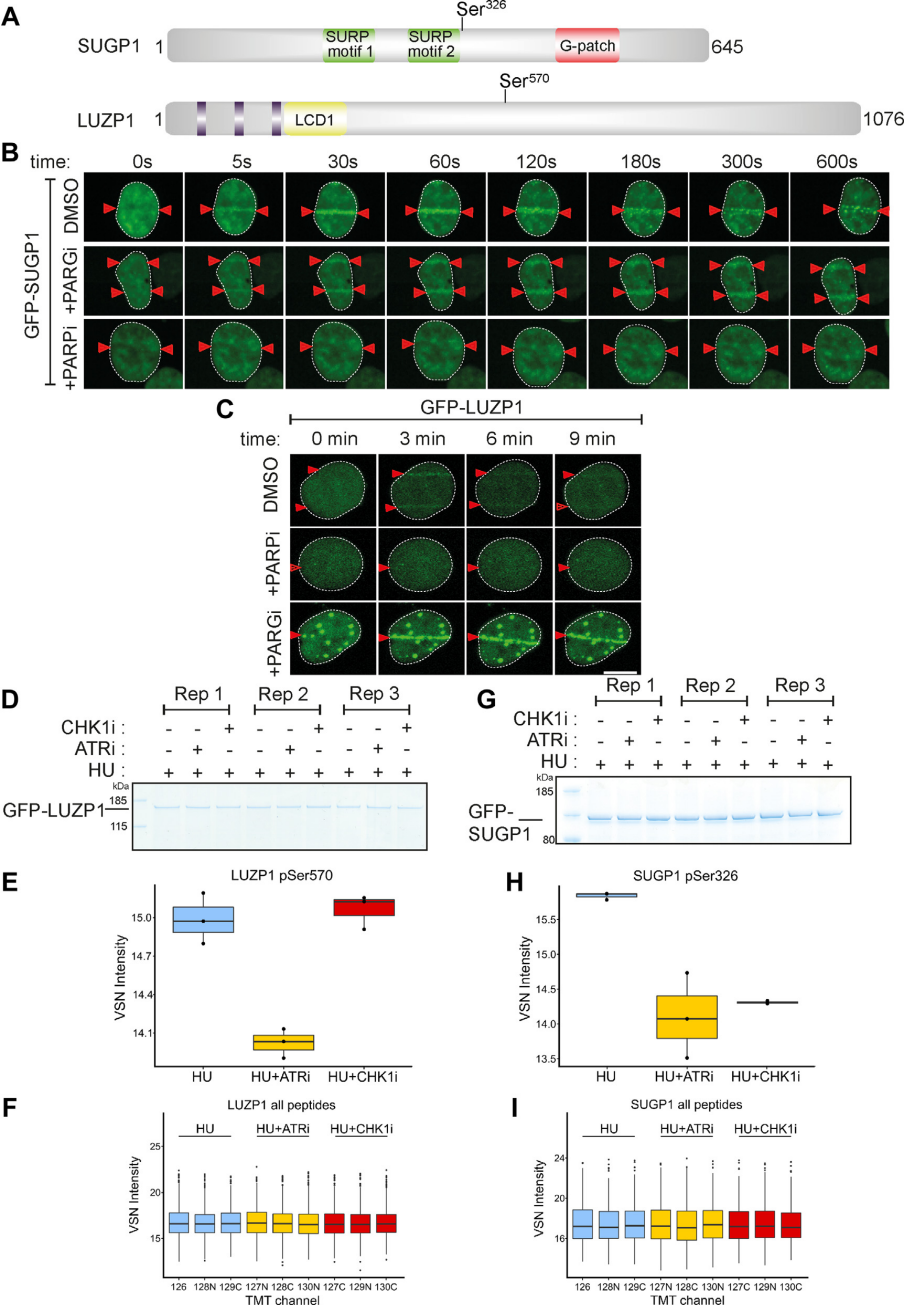
**Phosphorylation of SUGP1 and LUZP1 and recruitment to DNA damage sites**. *A*, schematic diagram showing the domain organization of SUGP1 and LUZP1. *B* and *C*, U-2 OS cells stably expressing GFP–SUGP1 (*B*) or GFP–LUZP1 (*C*) were preincubated with DMSO (mock), olaparib (5 μM; PARPi) or PDD00017273 (0.3 μM; PARGi) for 1 h prior to line micro–irradiation. Cells were live-imaged at the times indicated. *D*, U-2 OS cells were co-transfected with GFP-LUZP1. After 24 h cells were lysed, and cell extracts were subjected to immunoprecipitation with anti-GFP-agarose beads. Precipitates were subjected to SDS-PAGE and staining with Coomassie Brilliant Blue, and the bands corresponding to the GFP-tagged proteins were excised and processed for mass spectrometric detection of relevant phospho-peptides. Three independent co-transfection experiments were done for every condition (Rep = biological replicate). *E*, boxplots showing VSN–normalized intensity of phospho-peptides corresponding to LUZP1 pSer570 from the experiments in (*D*). *F*, boxplots of the VSN-adjusted TMT reporter ion intensities for all peptides for each TMT label in the case of GFP–LUZP1 from the experiment in (*D*). *G–I*, same as *D–F* except cells were transfected with GFP-SUGP1, and phosphorylation of Ser326 was analyzed in a similar manner. Mass spectrometry raw data was uploaded to ProteomeXchange *via* jPOSTrepo and can be accessed under the identifier PXD040476. Accompanying data analysis scripts and annotated spectra (66) are available from Zenodo under https://doi.org/10.5281/zenodo.10581706.

Several ATR targets of unknown function are recruited to micro-irradiation tracks, strongly suggesting roles in DDR. Some of these showed similarities in the kinetics of recruitment—for example, SUGP1 and LUZP1. SUGP1 is an uncharacterized protein containing two SURP motifs often found in proteins involved in pre-mRNA splicing (89, 90, 91), and a G-patch motif found in RNA binding proteins and in particular those with SURP motifs (92) (Fig. 4*A*). LUZP1 is largely uncharacterized but has been implicated recently in the control of primary cilia (Fig. 4*A*) (93, 94, 95). Recruitment of GFP-tagged forms of both of these proteins to micro-irradiation sites was rapid and transient (Fig. 4, *B* and *C*), reminiscent of proteins that bind poly–ADP ribose (PAR) chains generated by DNA damage–activated poly–ADP ribose polymerases (PARPs) (96). Consistent with this idea, recruitment of LUZP1 and SUGP1 was blocked by the PARP inhibitor olaparib; in contrast, retention time was prolonged by PDD00017273, an inhibitor of PARG (poly–ADP ribose glycohydrolase) which delays PAR degradation (Fig. 4, *B* and *C*) (97).

We next sought to validate ATR-dependent phosphorylation of these proteins by testing the phosphorylation of these proteins expressed in U-2 OS cells. LUZP1 Ser570 lies in the new consensus phosphomotif enriched among phosphorylation sites inhibited by berzosertib and gartisertib: pSRXXS (Fig. 2, *D* and *E*). Extracted ion chromatogram (XIC) analysis of tryptic phosphopeptides isolated from GFP-tagged LUZP1 (pSer570) confirmed that the HU-induced phosphorylation of this site is reduced by preincubating cells with berzosertib but not with the CHK1 inhibitor PF477736 (87) (Fig. 4, *D*–*F*). These data imply LUZP1 Ser570 is phosphorylated by a kinase downstream of ATR that is not CHK1 (Table 1). A similar XIC analysis of SUGP1 (pSer326) revealed that HU-induced phosphorylation of this site is reduced by preincubating cells with berzosertib or PF477736 (Fig. 4, *G*–*I*). Therefore, SUGP1 lies downstream of both ATR and CHK1 but pSer570 does not conform to the putative CHK1 RXXpS consensus motif, suggesting that a CHK1-activated kinase is responsible.

### Phospho-Dependent Interaction of SCAF1 with the RNAPII CTD

**Fig. 5:**
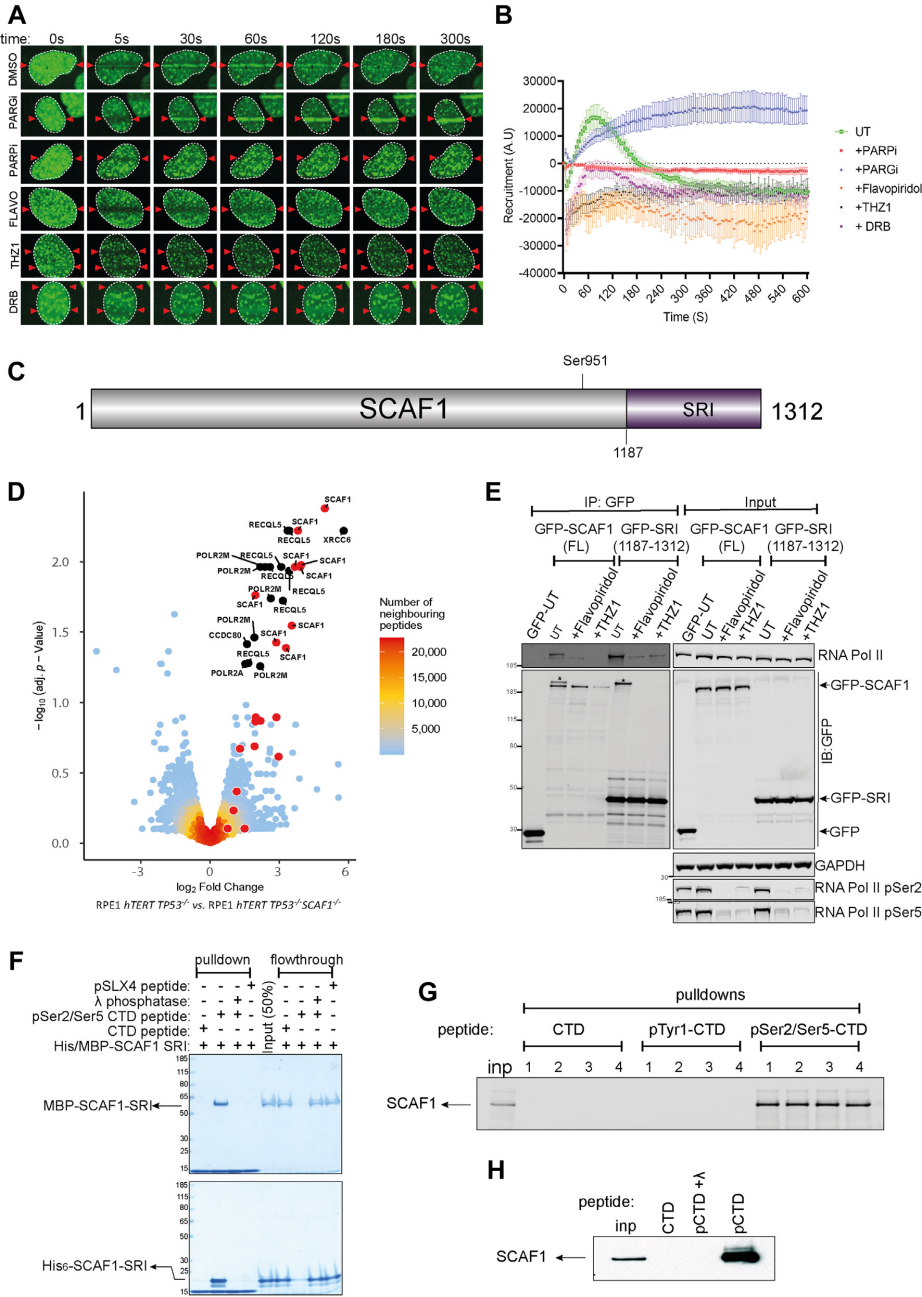
**SCAF1 interacts with the Ser2/Ser5-phosphorylated CTD of RNAPII**. *A*, BrdU-sensitized U-2 OS Flp-In T-REx cells stably expressing GFP-tagged SCAF1, pre–incubated with DMSO, olaparib (5 μM; PARPi), PDD00017273 (0.3 μM; PARGi), flavopiridol (10 μM), THZ-1 (10 μM) or DRB for 1 h were micro-irradiated with a 405 nm laser and imaged at the times indicated. *B*, same as (*A*), except that cells stably expressing GFP–SCAF1 were subjected to spot micro–irradiation (405 nm), and spot intensities were quantitated. Data represents the mean ± SEM of two independent experiments: >50 micro–irradiated cells per point. *C*, schematic diagram showing the domain organization of SCAF1. *D*, lysates of RPE1 *hTERT TP53*^*−/−*^ cells parental cells and RPE1 *hTERT TP53*^*−/−*^*SCAF1*^*−/−*^ cells were subjected to immunoprecipitation with in-house sheep anti-SCAF1 antibodies (3 biological replicates). Proteins were eluted from beads, trypsinized, and after TMT labeling, samples were pooled and injected on an UltiMate 3000 RSLCnano System coupled to an Orbitrap Fusion Lumos Tribrid Mass Spectrometer. A volcano plot representing SCAF1 interactors is shown. Dots in red (also with *White border* to differentiate from the peptide density) indicate SCAF1 peptides. Mass spectrometry raw data were uploaded to ProteomeXchange *via* jPOSTrepo with the identifier PXD041201. Data analysis scripts and annotated spectra (66) are available from Zenodo under https://doi.org/10.5281/zenodo.10581731. *E*, U-2 OS Flp-In T-REx cells expressing GFP-SCAF1, or GFP-SCAF1-SRI domain were subjected to immunoprecipitation with anti-GFP antibodies and precipitates were subjected to western blotting with the antibodies indicated (“IP-GFP”). Input cell extracts were analyzed in parallel. *F*, a biotinylated peptide containing three heptad repeats from the POL2RA CTD (CTD peptide), or the corresponding peptide that was phosphorylated at Ser2 and Ser5 in each heptad (pCTD) incubated or not with lambda phosphatase, was immobilized on streptavidin beads. A peptide corresponding to phospho-Ser1238 from SLX4 was used as a control. Beads were incubated with MBP-SCAF1-SRI or His6-tagged SRI expressed in bacteria. Beads were washed and subjected to SDS-PAGE and Coomassie staining. Both bead and supernatant “flowthrough” fractions are shown. *G*, the immobilized CTD and pS2/S5-CTD peptides were incubated with extracts of U-2 OS cells; after washing, precipitates were subjected to western blotting with SCAF1 antibodies. A CTD peptide where Tyr1 in each heptad was phosphorylated (pY1-CTD) was used as a control. *H*, same as (*E*), expect that the pS2/S5-CTD peptides were pre-treated or not with lambda phosphatase.

SCAF1 is a class iii ATR target of unknown function, but it was one of a range of genes identified previously in a genome-wide CRISPR-based screen for gene deletions that reverse the sensitivity of RPE-1 hTERT *BRCA1*^−/−^ *TP53*^−/−^ (*BRCA1*-KO cells) to PARP inhibitors (98). The fact that SCAF1 emerged for that screen and our ATR target screen suggested this may be a bona fide DDR regulator. Like SUGP1 and LUZP1, we found SCAF1 is recruited to sites of laser micro-irradiation in a transient manner; recruitment was blocked by olaparib, and retention time was prolonged by PDD00017273, demonstrating the dependence of poly-ADP ribose formation (Fig. 5, *A* and *B*). The cellular roles of SCAF1 are unknown, but it has a Set-Rpb1 interaction (SRI) domain towards the C-terminus (Fig. 5*C*) (99) which suggests interaction RNA polymerase (RNAP) II. The largest subunit of human RNAPII (POLR2A) has a C-terminal domain (CTD) bearing 52 tandem repeats of the consensus heptapeptide sequence Tyr^1^-Ser^2^-Pro^3^-Thr^4^-Ser^5^-Pro^6^-Ser^7^. CDK9/cyclin T preferentially phosphorylates the Ser2 sites of this sequence to promote transcriptional elongation; CDK7/cyclin H, a component of the general transcription factor (TFIIH), preferentially phosphorylates Ser5, which facilitates promoter clearance and transcriptional initiation (100, 101). SRI domains, in proteins such as the histone methyltransferase SETD2 and the helicase RECQL5 (implicated in DNA replication, transcription and repair), interact with the Ser2/Ser5-phosphorylated form of RNAPII CTD (99, 102, 103, 104, 105). Furthermore, a recombinant fragment of the SCAF1 SRI domain was reported to interact with recombinant CTD (106). To test if SCAF1 interacts with RNAPII in cells, endogenous SCAF1 was immunoprecipitated from RPE-1 cells using antibodies generated in-house, and *SCAF1* knockout (KO) RPE-1 cells were used as control. Quantitative mass spectrometric analysis identified a range of proteins that were much higher in abundance in SCAF1 precipitates from parental cells than from SCAF-KO cells (Fig. 5*D*). Besides SCAF1 itself, this included POLR2A and several other subunits of RNAPII as well as RECQL5, known to associate with RNAPII through its SRI domain (104). We speculate the interaction of RECQL5 is indirect through RNAPII-CTD.

We next set out to test if the interaction of SCAF1 with RNAPII is mediated by the phospho-dependent interaction of the SCAF1 SRI domain with the Ser2/Ser5-phosphorylated form of POLR2A CTD. As shown in Figure 5*E*, brief exposure of cells to flavopiridol or THZ1 which inhibits CDK7 and CDK9-dependent phosphorylation of POLR2A Ser2/Ser5 (107, 108, 109) severely reduces the association of GFP-SCAF1 or GFP-SRI with POLR2A. To investigate further, synthetic peptides containing three heptad repeats from the POL2RA CTD, phosphorylated (“pCTD peptide”) at Ser2 and Ser5 of each heptad, bearing biotin at the N-terminus, were immobilized on streptavidin beads; the non-phosphorylated peptide was used as control (“CTD peptide”). Pulldown experiments revealed that recombinant MBP-tagged or His_6_-tagged forms of the SCAF1 SRI domain expressed in bacteria were efficiently retrieved by the pCTD beads but not by the CTD beads or beads bearing a control SLX4 phosphopeptide (Fig. 5*F*). Furthermore, lambda phosphatase pre-treatment of the pCTD beads prevented retrieval of the SCAF1 SRI domain. We also found that the pSer2/Ser5-CTD beads, but not beads bearing the CTD peptide or the CTD peptide where Tyr1 in each of the three heptads was phosphorylated, could retrieve endogenous SCAF1 from cell extracts (Fig. 5*G*). Pre-treatment of the pSer2/Ser5 CTD beads with lambda phosphatase prevented retrieval of SCAF1 from cell extract (Fig. 5*H*). We also tested if the inhibitors that block its interaction with RNAPII CTD (Fig. 5*E*) also affect the recruitment of SCAF1 to DNA damage sites. As shown in Figure 5, *A* and *B*, the recruitment of SCAF1 to sites of laser micro-irradiation is inhibited by THZ-1 and flavopiridol, and in fact, SCAF1 appears to be excluded from DNA damage tracks when cells are exposed to these drugs. SCAF1 recruitment is also inhibited by DRB (5,6–dichloro–1–β-D-ribofuranosylbenzimidazole) which inhibits CDK9 (110) (Fig. 5, *A* and *B*). In contrast, flavopiridol, THZ-1, and DRB do not affect the recruitment of the PAR-responsive protein XRCC1 (Supplemental Fig. S3). Taken together the data above show that the SRI domain of SCAF1 interacts with the Ser2/Ser5-phosphorylated form of RNAPII, and this interaction may facilitate SCAF1 recruitment to DNA damage sites.

### SCAF1 Deficiency in *BRCA1*^−/−^ Cells Causes Partial Restoration of HR

**Fig. 6:**
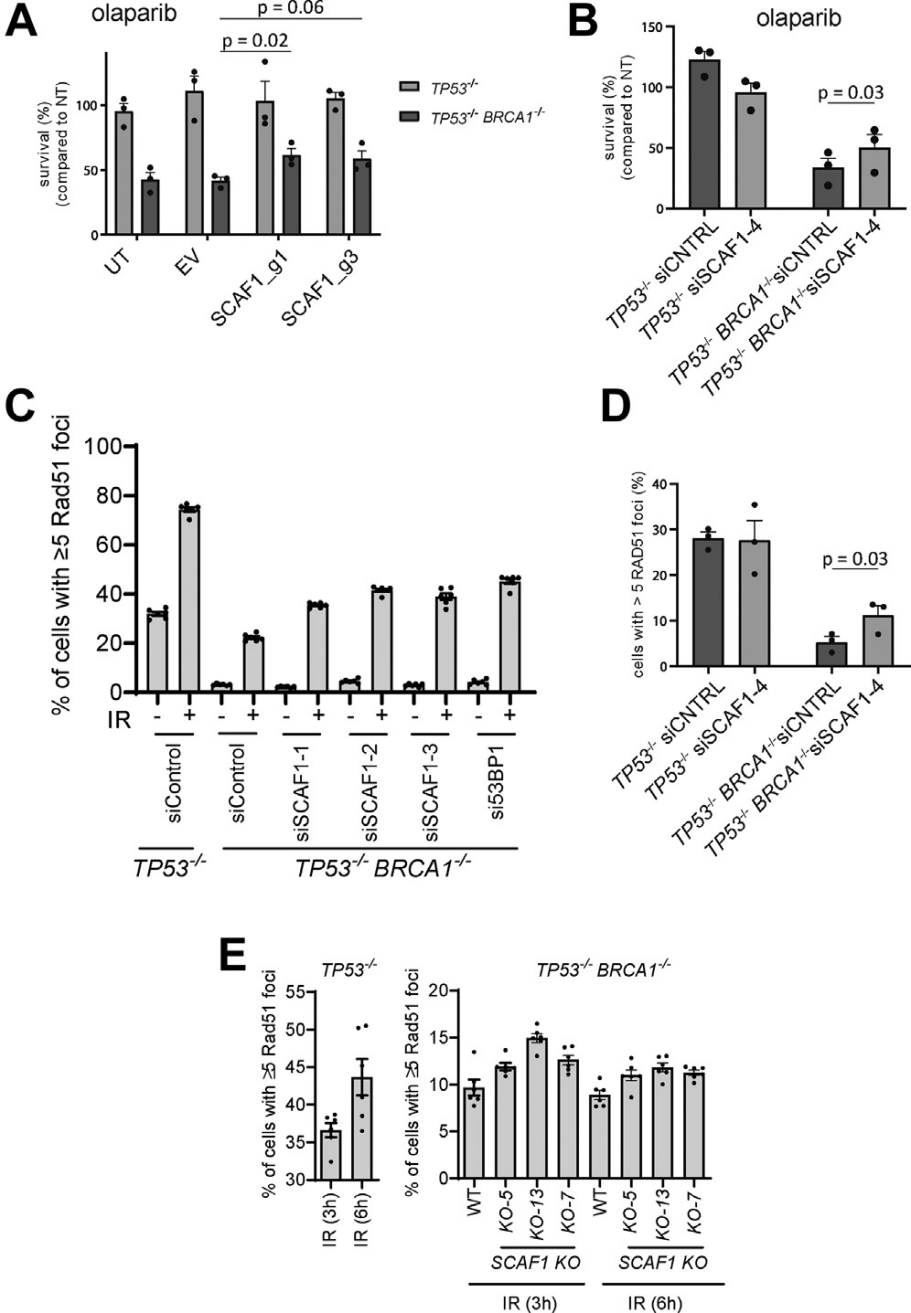
**SCAF1 is a new genome maintenance factor**. *A*, the cell lines indicated were virally transduced with sgRNAs targeting *SCAF1* and clonogenic survival was assessed in the presence or absence of olaparib (16 nM) on the pool of transduced cells. sgRNA targeting efficiency was assessed using genomic PCR amplification of the targeted locus and TIDE analysis. Editing efficiencies were >50% for all conditions (see Experimental Procedures). Data is represented as mean +SEM (n = 3), *p*-values are obtained using a two-tailed *t* test. *B*, same as (*A*), but here SCAF1 was depleted using siRNA transfection prior to clonogenic survival. Data is represented as mean +SEM (n = 3), *p*-value is obtained using a two-tailed *t* test. *C*, the cell lines indicated were transfected with the siRNAs indicated and after 24 h cells were exposed to IR (10 Gy). Cells were allowed to recover for 3 h, fixed and the proportion of RAD51 cells with greater than five foci was assessed by immunofluorescence. Data was acquired with a high-content screening station ScanR. Data from three independent experiments were combined; data are represented as mean ± SEM. *D*, Same as (*C*), expect that the experiment was performed by SN. Data is represented as mean +SEM (n = 3), *p*-value is obtained using a two-tailed *t* test. *E*, same as (*C*)*,* except that the indicated cell lines were used. EV, empty vector; UT, untreated.

SCAF1 was one of a range of genes identified previously in a genome-wide CRISPR-based screen for gene deletions that reverse the sensitivity of RPE-1 hTERT *BRCA1*^−/−^ *TP53*^−/−^ (*BRCA1*-KO cells) to PARP inhibitors (98). We first set out to validate these data. As shown in Figure 6*A*, two different small guide (sg) RNAs targeting SCAF1 caused a modest but reproducible increase in the resistance of *BRCA1*-KO cells, but not the parental *TP53*^−/−^ cells, to the PARP inhibitor olaparib. A SCAF1-specific siRNA had a similar effect (Fig. 6*B* and Supplemental Fig. S4*A*). The reversal of olaparib sensitivity suggests that depleting SCAF1 may restore HR in *BRCA1*-KO cells, and we set out to investigate this possibility by visualizing the recruitment of the RAD51 recombinase to IR-induced DSB, known be defective in *BRCA1*-KO cells (111). We found that siRNA-mediated depletion of SCAF1 using a series of individual siRNAs increased the proportion of *BRCA1*-KO cells with RAD51 foci with similar effect size to depletion of 53BP1 (Fig. 6, *C* and *D* and Supplemental Fig. S4, *B* and *C*), which is a well-known inhibitor of HR in *BRCA1*-deficient cells (112, 113). We used genome editing to generate clonal *SCAF1* gene knockouts in *BRCA1*-KO cells (Fig. 6*E*) and found that *SCAF1* deletion increased the proportion of cells with greater than five IR-induced RAD51 foci (Fig. 6*E*). Together these data indicate that SCAF1 suppresses HR in *BRCA1*-deficient cells.

The early steps of homologous recombination involve the resection of DNA ends (5′–3′ degradation) leading to a stretch of 3′-ended single-stranded DNA which is then repaired using the sister chromatid as a DNA template. It was shown previously 53BP1 and shieldin suppress HR in *BRCA1*-KO cells by suppressing DNA end resection and we therefore analyzed the impact of SCAF1 on DNA resection. Resected DNA exposes BrdU-incorporated regions of single-stranded DNA, and immunofluorescence analysis using BrdU antibodies under non-denaturing conditions serves as a readout for DSB resection. We observed that deletion of *SCAF1* in *BRCA1*-KO cells leads to increased levels of single-stranded DNA (Supplemental Fig. S4, *D* and *E*). To further validate these results, we monitored phosphorylation of RPA32 at Ser 4/Ser 8, a surrogate marker of DNA resection. Surprisingly, the levels of RPA32 phosphorylation in *BRCA1*-KO *SCAF1*-KO cells were not noticeably different from single *BRCA1*-KO cells monitored by western blotting or immunofluorescence (Supplemental Fig. S4, *F* and *G*). These data suggest that, while increasing single-stranded DNA in BRCA1-KO cells, other mechanisms rather than DNA end resection may provide PARPi resistance after *SCAF1* deletion.

## Discussion

In this study we carried out parallel phosphoproteomic screens to identify proteins that are sensitive to the ATR inhibitors berzosertib or gartisertib. We identified 176 phosphorylation sites in 144 proteins whose phosphorylation is reduced by both drugs, which corresponds to around 45% of the total number of phospho-sites affected by each inhibitor (Fig. 2*C*). These overlapping targets are highly likely to be *bona fide* targets of the ATR pathway, although not all of them are direct ATR targets. A range of phosphoproteomic screens have already been reported using a diverse range of conditions – using a variety of approaches involving a range of cell lines treated with different ATR inhibitors including VE-821(50–52), AZ20 (46), AZD6738 (48), BAY1895344 (47) or using cells lines defective in ATR (49) or ATR activators (45). A detailed comparison of the ATR targets identified in these screens and our screen is beyond the scope of this manuscript, but it is important to point out that the phosphorylation sites focussed on in this study in TLK2, FANCJ, SUGP1, LUZP1, and SCAF1—had not been identified in previous screens and they represent new ATR targets. While this manuscript was under review, DHX9 was reported to be phosphorylated on the Ser321 site identified in the current study, providing further validation of our screen (88).

In downregulated phospho-sites, motif analysis showed that around half the sites downregulated by both berzosertib and gartisertib conform to the S/T-Q consensus motif typically phosphorylated by ATR and related kinases such as ATM and DNA-PK. The sites not conforming to the S/T-Q motif show enrichment for the motif pSRXXS (Fig. 2, *D* and *E*). LUZP1 Ser570, for example, conforms to the pSRXXS motif, and we showed that its phosphorylation is dependent on ATR but not CHK1 (Fig. 4, *D*–*F*). We speculate that phosphorylation of these motifs requires an ATR-activated kinase other than CHK1. Candidate kinases include those belonging to the SRPK family of kinases, which can phosphorylate serine residues that lie in SR motifs (114), and this will be interesting to investigate especially as these kinases have been linked to genome stability (115, 116). It could be argued that the pSRXXS motif enrichment is caused by missed (tryptic) cleavages at the CHK1 consensus sequence (RXXpS). However, phospho-motif searching was performed exclusively with singly phosphorylated peptides showing a false localization rate (FLR) of the phosphorylation site of 1% (MaxQuant PTM-Score probability ≥0.994) (117). Using singly phosphorylated peptides only specifically enables the assignment of a regulated phosphorylation site to a defined residue. Further, filtering phospho-site localizations with a MaxQuant PTM-Score probability cut-off of ≥0.994 (1% FLR), helped to exclude mislocalization of a CHK1-typical SRXXpS phosphorylation site. This idea is strengthened by the observation that out of the 38 unique peptides in our dataset that contain an ATR inhibitor-sensitive phosphorylation site within a pSRXXS motif, 30 peptides were cleaved by trypsin after the arginine preceding the second serine, yielding a pSR peptide C-terminus, thus a mislocalization of the phospho-site giving an artefactual pSRXXS instead of an SRXXpS is highly unlikely. Intriguingly, enrichment of non-overlapping phospho-motifs specific to each individual inhibitor was observed: YX[pS/pT] for berzosertib and pSXXXTP YXS/T for gartisertib. The most likely explanation is that reduced phosphorylation of these motifs is caused by off-target effects of the inhibitors, but this remains to be investigated.

The first of the three classes of ATR targets we defined contain phosphorylation sites that are highly sensitive to ATRi and these are prime candidates for new ATR biomarkers. The phospho-site most sensitive to ATRi is Ser597 of RAP80 (Fig. 2*F*). ATR was reported to phosphorylate RAP80 at Ser205 (pSQ) after UV exposure (118), but phosphorylation of Ser597, which does not conform to the classical consensus motif for ATR or CHK1, has not been reported previously. In this light, it could be argued that a new ATR biomarker for use in clinical trials should correspond to a classical pS/pT-Q motif. PMS2 pSer603, RAD18 pSer368, and claspin pSer921 all fit this description (Fig. 2*F*) and therefore represent potentially powerful new biomarkers, especially in trials where ATRi is used as monotherapy. The second class of new ATR target sites lie in proteins already linked to DDR, and most of these sites lie in pS/pT-Q motifs including FANCJ (Thr113 and Ser226), MCM9 (Ser663) and SMC1 (Ser358) and EXO1 (Ser714) (Supplemental Table S5). We validated the phosphorylation of DHX9 Ser321, which lies close to the helicase domain, and it will be interesting to test if ATR regulates DHX9 activity (Fig. 3, *A* and *C*). Intriguingly, DHX9 is unusual in that it is excluded from DNA damage sites (Fig. 3*F*); this will be interesting to investigate further. A small number of other phosphorylation sites in this class 2 hits lie in functionally annotated domains: for example, within the FANCJ helicase domain (Thr113 and Ser226) and the TLK2 kinase domain (Ser686). Neither protein is known to be regulated by ATR; it will be interesting to test if the catalytic activity of these enzymes is altered in a phospho-dependent manner in response to replication stress or DNA damage. Class 2 targets also include proteins linked to cellular processes known to be controlled by ATR. For example, TERF2IP (RAP1), binds to the shelterin component TRF2 and plays multiple roles in telomere protein and length maintenance (119). It will be interesting to test the functional impact on telomere biology of mutating the ATR-dependent phosphorylation site in RAP1.

The third class of new ATR target sites lies in proteins not previously known to be ATR targets or linked to DDR or RS responses, and they all represent candidate DDR factors. The cellular roles of some of these proteins is either unclear or only beginning to be understood. One of these proteins, SUGP1 is also one of the proteins whose phosphorylation is most sensitive to ATRi. SUGP1 is phosphorylated in an ATR- and CHK1-dependent manner on Ser326 and is recruited to DNA damage sites in a manner that depends on PAR synthesis. The role of SUGP1 is only beginning to be understood, but it was recently identified as a binding partner of SF3B1, a spliceosome component that is commonly mutated in cancers and myelodysplastic syndrome. Most of the disease-causing SF3B1 mutations lie in the HEAT repeats, and some cluster in a small area within these repeats including the most frequently mutated residue Lys700 (120). This mutation was shown to disrupt interaction with SUGP1 selectively, and deletion of SUGP1 recapitulates the splicing errors seen in cells expressing the SF3B1 K700 mutation (121). There are already intriguing links between SF3B1 and the DDR—for example, SF3B1-mutated cells accumulate DNA damage and R-loops and show reduced apoptotic responses to DNA damage (122, 123). Moreover, SF3B1-mutated MDS cells show increased levels of RS and ATR activation (124). Therefore, it is clear that SUGP1, in complex with SF3B1, is likely to be a key player in DDR. It is possible that SUGP1-SF3B1 impacts DNA damage responses through controlling the splicing of DDR factors, as was shown recently for DYNLL1 (125). However, the observation that SUGP1 is recruited to DNA damage sites argues that it may have a more direct role, perhaps in collaboration with SF3B1. It will be important to study the regulation of this complex by ATR-dependent phosphorylation. The Ser326 site in SUGP1 conforms to neither the ATR or CHK1 consensus motifs and so we speculate that a CHK1-activated kinase is responsible, but no such kinase is yet known.

*SCAF1* was one of a range of genes identified in a screen for gene deletions that render *BRCA1*-knockout cells resistant to PARP inhibitors (98). Other positives identified in this screen included *53BP1* and components of the shieldin complex; deleting these factors partly restored DNA end resection, RAD51 foci, and HR in *BRCA1*-KO cells. Our identification of SCAF1 as an ATR target reinforced the notion that it may regulate genome stability which we explored further. In this light, SCAF1 depletion or deletion causes a modest reversal of the olaparib sensitivity of *BRCA1*-knockout cells, but also causes a modest restoration of HR judged by RAD51 foci (Fig. 6). At present the mechanistic basis for this rescue is unclear. Factors such as shieldin that emerged from the same genetic screen inhibit HR in *BRCA1*-KO cells by suppressing DNA end resection. We found a modest increase in ssDNA levels after IR in *SCAF1*-deleted *BRCA1*-KO cells, judged by BrdU foci, suggesting increased DNA end resection. However, no corresponding increase in levels of phospho-RPA, an alternative readout of resection, was observed. This discrepancy may be explained by the modest effect size of SCAF1 deletion in restoring RAD51 foci and reversing olaparib sensitivity *BRCA1*-KO cell. The increase in resection may not have reached the threshold for triggering RPA phosphorylation. It is interesting to note that deleting *SCAF1* in BRCA1-proficient hTERT *TP53*^−/−^ RPE-1 cells appeared to decrease the levels of ssDNA after IR—the opposite to *BRCA1*-deficient cells. The basis for this surprising observation is not yet clear, but it may be that SCAF1 has different roles in different genetic contexts. Whatever the case, it will be interesting to explore the mechanisms underlying the impact of SCAF1 on HR in *BRCA1*-KO cells, and a key question concerns the relevance of the phospho-dependent interaction of SCAF1 with RNAPII. Given this association, it is possible that SCAF1 impacts HR by influencing the expression - or splicing - of one or more genes involved in the control of HR. However, our finding that SCAF1 localizes to DNA damage sites suggests a more direct role. It is possible that SCAF1-regulated transcriptional processes impact HR, independent of changes in gene expression. Understanding the links between SCAF1 association with RNAPII CTD and its role in inhibiting HR, at least in *BRCA1*-knockout cells will be important.

## Data Availability

All the data are available in the main text or supplementary materials. All mass spectrometry raw data, MaxQuant search settings and output files were uploaded to PRIDE (PXD041250) or jPOSTrepo (PXD040469, PXD040476, PXD041201, PXD050953 and PXD050954) and all can be downloaded *via* ProteomeXchange with their respective identifier. Data analysis scripts, and annotated spectra (66) can be downloaded from Zenodo under https://doi.org/10.5281/zenodo.10581948, https://doi.org/10.5281/zenodo.10581706, https://doi.org/10.5281/zenodo.10581731, https://doi.org/10.5281/zenodo.10882997 and https://doi.org/10.5281/zenodo.10850046.

## Supplemental data

This article contains supplementary information. Supplemental data, materials and methods including references were furnished in the supplementary information.

## Conflict of interest

The authors declare that they have no conflicts of interest with the contents of this article.
