# Supplementary material for: Chemo-Phosphoproteomic Profiling with ATR Inhibitors Berzosertib and Gartisertib Uncovers New Biomarkers and DNA Damage Response Regulators
Source: Mol Cell Proteomics. 2024 Jun 15;23(8):100802. doi: 10.1016/j.mcpro.2024.100802 (PMC11338954; doi:10.1016/j.mcpro.2024.100802)
Supplement: Supplementary information [file mmc10.pdf]

Chemo-phosphoproteomic profiling with ATR inhibitors berzosertib and gartisertib uncovers new biomarkers and DNA damage response regulators.

Authors: Rathan Jadav[1,9], Florian Weiland[1,3,9], Sylvie M. Noordermeer[4,5], Thomas Carroll[1], Yuandi Gao[6], Jianming Wang[1], Houjiang Zhou[1,7], Frederic Lamoliatte[1], Rachel Toth[1], Thomas Macartney[1], Fiona Brown[1], C. James Hastie[1], Constance Alabert[2], Haico van Attikum[6], Frank Zenke[8], Jean-Yves Masson[6] & John Rouse[1*]

List of Supplementary Data

Fig. S1. Quantitative phosphoproteomic screening workflow

Fig. S2. Analysis of Gene Ontology and protein recruitment

Fig. S3 Transcriptional inhibitors do not affect XRCC1 recruitment to DNA damage sites.

Fig. S4 SCAF1 depletion restores RAD1 foci in BRCA1-knockout cells.

Fig. S5 Protein interaction network relating to phosphopeptides downregulated after gartisertib

Fig. S6 Protein interaction network relating to phosphopeptides downregulated after berzosertib

Fig. S7 Phosphorylation sites upregulated after ATR inhibitor treatment

Fig. S8 Protein interaction network relating to phosphopeptides commonly upregulated after gartisertib & berzosertib

Fig. S9 Representative annotated spectrum of the DHX9 phosphopeptide containing pSer321

Table S1. Mass spectrometry summary statistics.

Table S2. Phosphoproteomic dataset: U-2 OS cells, HU ± berzosertib.

Table S3. Phosphoproteomic dataset: U-2 OS cells, HU ± gartisertib

Table S4. Overlap between hits in Table S2 (HU ± berzosertib) and Table S3 (HU ± gartisertib).

Table S5. New ATR targets already implicated in cell responses to DNA damage or replication stress.

Table S6. New ATR targets not previously implicated in cell responses to DNA damage or replication stress.

Table S7. Phosphorylation sites higher in abundance after ATR inhibitor treatment

Table S8. Proteins with phosphorylation sites higher in abundance after ATR inhibitor treatment

Table S9. Lists of reagents used in this study

SUPPLEMENTARY MATERIALS AND METHODS

Phosphoproteomic screening

Sample preparation for phosphoproteomic analysis

Five 150 mm plates were seeded with U-2 OS cells at 30% confluency and synchronised to enrich cells in S phase as described in the method section. Cells were mock treated or treated with 1 µM ATR inhibitors (berzosertib or gartisertib) followed by treatment with 1 mM hydroxyurea for 30 min. Post drug treatment, cells were harvested by trypsinisation and washed once with ice cold PBS. To the cell pellets 1.5 mL ice cold TCA-Acetone solution (20 % (v/v) TCA, 80% (v/v) acetone, 0.2% DTT (wt/v)) was added, cells were vortexed vigorously to disperse the pellet and kept overnight at -20°C. After 24 h, samples were centrifuged at 20,000 g at -9°C for 20 min, and the supernatants was discarded. Pellets were washed once with 2 mL ice cold 80% (v/v) acetone, vortexed vigorously, centrifuged again at 20,000 g at -9°C for 20 min. Carefully removed the supernatant and pellets were air dried for 10 min at room temperature. Cell pellets from each plate were then lysed in 350 µL of 8 M urea, 50 mM AmBiC, 1% (v/v) phosphatase inhibitor cocktail-2, 0.1% (v/v) microcystin-LR, pH 8. Samples were mixed thoroughly by pipetting up and down and incubated at room temperature for 15 min. Post incubation samples were sonicated using a Bioruptor sonicator at high amplitude for 10 cycles (30 sec on/30 sec off). Lysates were clarified by centrifugation at 17,000 g for 10 min and samples were stored at -80°C until further use for mass spectrometric analysis. Five independent biological replicates per condition were prepared on different days using the two different ATR inhibitors berzosertib and gartisertib. Protein concentrations were determined using BCA assay.

A total of 3.5 mg total protein from each sample was taken and volume was equalized using 8M urea (Cat. No. 1.08487.0500, Merck, Darmstadt, Germany), 50mM ammonium bicarbonate

(AmBiC, Cat. No. 09830, MilliporeSigma, St. Louis, Missouri, US), 1% (v/v) Phosphatase Inhibitor Cocktail 2 (Cat. No. P5726, MilliporeSigma) and 0.1% (v/v) Microcystin-LR (Cat. No. 33893, MilliporeSigma). Proteins were then reduced and alkylated with 10mM tris(2-carboxyethyl) phosphine (TCEP, Cat. No. C4706, MilliporeSigma), 25mM Chloroacetamide (Cat. No. C0267, MilliporeSigma) in the dark for 45 minutes at room-temperature (RT). Afterwards, the samples were diluted with 50mM AmBiC (MilliporeSigma) to a concentration of 2M Urea. Trypsin/LysC (Cat. No. V5071, Promega, Madison, Wisconsin, US) was added at a 1:50 enzyme:protein ratio and the samples were incubated at 37°C overnight. Samples were acidified by adding trifluoroacetic acid (TFA, Cat. No. 1.08262.0025, Merck) to a concentration of 1% (v/v) and centrifuged at 3,270 x g for 15 minutes at RT. Supernatant was desalted by C_{18} chromatography using Sep-Pak cartridges (830 mg, Cat. No. WAT023635, Waters, Milford, Massachusetts, US), eluted peptides were snap frozen and freeze dried overnight.

Phosphopeptide enrichment and TMT labelling

Samples were reconstituted in 300 µL 250mM lactic acid (Cat. No. 252476, MilliporeSigma), 70% (v/v) Acetonitrile (ACN, Cat. No. 1.00029.1000, Merck), 3% (v/v) TFA (Merck). Peptides were incubated with TiO_2 beads (Cat. No. 5010-21315, GL Sciences, Tokyo, Japan) at a ratio of 1mg peptide: 4mg beads. Beads were washed with 250mM lactic acid, 70% (v/v) ACN, 3% (v/v) TFA, then with 70% (v/v) ACN, 3% TFA and as last step with 0.1% (v/v) TFA. Phosphopeptides were eluted from the beads using 1% (v/v) NH_4OH (in case of samples derived from berzosertib experimental series: 50% (v/v) ACN (Merck) was added to elution buffer as well), snap frozen and freeze dried overnight. This phosphopeptide enrichment procedure was repeated for a total of 2 times. Phosphopeptide enrichment was checked via LC-MS/MS and an average of 8.66% (gartisertib) and 10.93% (berzosertib) of non-phosphorylated peptides were detected. Subsequently, the phosphopeptides were labelled via tandem mass tags (TMT10plex, Cat. No. 90113, Thermo Fisher Scientific, Waltham, Massachusetts, US). Freeze dried peptides were

reconstituted in 100mM Triethylammonium bicarbonate (TEAB, Cat. No. 90114, Thermo Fisher Scientific). TMT labels were resuspended in anhydrous ACN (Cat. No. 271004, MilliporeSigma), added to the respective samples and incubated 2h at RT. In case of the (HU vs. HU + gartisertib) experimental series, the HU group was labelled with TMT126-TMT128C, the inhibitor treatment group was labelled with TMT129N-TMT131. In the experimental series (HU vs HU + berzosertib), the inhibitor treatment group was labelled with TMT126-TMT128C, while the HU group was labelled with TMT129N-TMT131. An aliquot was taken (the remainder samples were interim stored at -80°C) and labelling efficiency was checked via LC-MS/MS. Peptide N-termini were detected as labelled in 96.8% (gartisertib) or 87.2% (berzosertib) of all peptides, and lysines were detected as labelled in 97.8% (gartisertib) or 85.1% (berzosertib) of all cases. The labelling reaction of the remainder samples was stopped using hydroxylamine (Cat. No. 467804, MilliporeSigma). Afterwards, the samples were pooled, snap frozen and freeze dried.

Sample prefractionation using high pH reverse phase liquid chromatography

The pooled sample was fractionated using high pH reverse phase liquid chromatography. Peptides were reconstituted in 200 µL 10mM ammonium formate (Cat. No. 70221, MilliporeSigma) pH 10 and separated on a C_{18} column (4.6 x 250 mm, 3.5µm particle size, Cat. No. 186003570, Waters) using a two-component buffer system. Buffer A consisted of 10mM ammonium formate, pH 10 while Buffer B consisted of 80% acetonitrile and 10mM ammonium formate, pH10. Peptide separation was achieved using a 75min gradient with following settings: 0-5.5 minutes 3% (v/v) Buffer B (flowrate: 275µl/min); 5.5-10 minutes linear gradient to 10% (v/v) Buffer B (flowrate: 569 µl/min); 10-45 minutes linear gradient to 35% (v/v) Buffer B, 45-55 minutes linear gradient to 50% (v/v) Buffer B; 55-65 minutes linear gradient to 80% (v/v) Buffer B; 65-67.5 minutes linear gradient to 100% Buffer B; 67.5-70 minutes 100% Buffer B; 70-70.5 minutes gradient to 3% (v/v) Buffer B; 70.5-75 minutes 3% (v/v) Buffer B. 75 fractions were

collected, concatenated into 20 fractions, snap frozen, freeze dried and stored at -80°C until further usage.

LC-MS/MS

Before LC-MS/MS analysis, samples were reconstituted in 2% (v/v) ACN (Merck), 0.1% formic acid (FA, Cat. No. 5.33002.0050, Merck). LC-MS/MS was performed using a Thermo Dionex Ultimate 3000RLSC system coupled to an Orbitrap Fusion Tribrid mass spectrometer (Thermo Fisher). Samples were injected in triplicates onto a C_{18} trap column (Acclaim™ PepMap™ 100, Cat. No. DX164564, Thermo Fisher) and washed for 5 minutes using 3% (v/v) ACN, 0.5% (v/v) TFA at a flowrate of 5 µL/min. The column oven was set to 45°C. Peptides were separated on a C_{18} column (EASY-Spray™, 50 cm length, Cat. No. 03-251-874, Thermo Fisher) using a segmented linear gradient applying a two-buffer system with a total run-time of 180 minutes at a flowrate of 300 nL/min. Buffer A consisted of 3% (v/v) ACN, 0.1% (v/v) FA, Buffer B of 80% ACN, 0.08% (v/v) FA. During the first 145 minutes Buffer B percentage increased from 5 to 25%, followed by an increase to 35% B until minute 155, then increase to 95% B until minute 160 with isocratic elution until minute 165, this was followed by a decrease in Buffer B to 3% over 30 seconds with isocratic column wash until minute 180. Eluted peptides were injected into the Orbitrap MS. The ion source spray voltage was operated in positive mode and set to 2000 V. Ion transfer tube was heated to 275 °C. The mass spectrometer was operated in data-dependent Top20 mode. MS full scan was performed using the Orbitrap detector, detecting positively charged ions between 375-1500 *m/z* at a resolution of 120K at 200 *m/z*. AGC target was set to 4×10^5 with a maximal ion injection time of 50 ms. For MS2 scan, precursor ions had to exhibit the following properties: Intensity above 2×10^4, charge states between 2 and 7, while exhibiting an isotopic distribution expected for a peptide. Precursor ions were isolated using a width of 1.6 *m/z* and fragmented by higher-energy collisional dissociation (HCD) with 37% normalized collision energy. AGC target was set to 5×10^4. In case of samples from gartisertib

experimental series the maximum injection time was set to 120 ms, while for samples from the berzosertib series this was set to 200 ms. MS/MS spectra were recorded in the Orbitrap detector at a resolution of 50K (at 200 *m/z*). To avoid repeated fragmentation of the same precursor ion species, dynamic exclusion was set to 40 seconds with a mass tolerance of 10 ppm.

Data analysis global phosphoproteomics

Mass spectrometry raw data was searched against a *homo sapiens* FASTA file (42,326 entries including protein isoforms, downloaded from www.uniprot.org on 5th April 2018) (1) using MaxQuant (Version 1.6.2.3) (2). Default search settings were applied, as digestion enzyme Trypsin/P was set and a maximum 2 missed cleavages was allowed. As fixed modifications the carbamidomethylation of cysteine and as variable modifications the oxidation of methionine, acetylation of the protein N-terminus was set. Additionally, the following non-default variable peptide modifications were set: Phosphorylation of serine, threonine and tyrosine, deamidation of asparagine. Mass tolerance for the precursor ions for the first search was set to 20 ppm, the main search tolerance was 4.5ppm. The MS/MS tolerance was set to 20 ppm. Peptide false discovery rate (FDR) and protein FDR was set to 5%. All mass spectrometry raw data, the MaxQuant search parameters and output files were uploaded to jPOSTrepo (3) and can be downloaded via ProteomeXchange (4) with the identifier PXD040469. Results were analysed using an in-house developed R (version 4.1.1) (5) computational pipeline, based on modified scripts applied in (6). In brief, within each fraction the peptide intensities were averaged. Data were normalized using variance stabilizing normalization (VSN) (7, 8) and statistically tested using linear models for microarrays (*limma*) (9, 10). A peptide was regarded as differentially abundant between the two conditions if the adjusted p-value was ≤ 0.05. Singly phosphorylated peptides, lower abundant under inhibitor treatment, exhibiting a phosphorylation PTM score probability ≥ 0.994 (corresponding to a false localization rate of 1% (11)) underwent

phosphorylation sequence motif analysis using rmotifx (12). GO term analysis was based on a modified version of a previous R script file (6), applying a Fisher's exact test. For this, the unique leading razor protein names connected to phosphopeptides lower abundant under inhibitor treatment were used as foreground. The unique leading razor protein names of the whole dataset were used as background. Resulting p-values were converted into q-values (13) and GO terms with $q \leq 0.01$ and at least 10 protein hits were regarded as enriched in the foreground.

Peptide and protein metadata were mined from following databases: Protein interaction partners: BIOGRID (version 4.4.217) (14), phosphorylation sites: PhosphoSitePlus® (version 122022, downloaded from www.phosphosite.org on the 20th December 2022) (15), Gene ontology terms: downloaded from www.uniprot.org on 6th January 2023, Protein modifications dataset was downloaded from www.uniprot.org on 6th January 2023. Protein domains data were extracted from Ensembl (16), Interpro (17), Prosite (18) and Pfam (19) using the R biomaRt library (20, 21). List of kinases (pkinfam) was downloaded on 06/05/2020 from https://www.uniprot.org/docs/pkinfam, list of E3 ligases from https://hpcwebapps.cit.nih.gov/ESBL/Database/E3-ligases/ on 28/08/2019 (22), and the list of deubiquitylases (dubs.txt) was downloaded 06/05/2020 from https://www.genenames.org/data/genegroup/#!/group/996. Protein-protein interaction network analysis (Figure S5 and S6) was conducted using STRING-db (23, 24).

Additional R libraries used were: miscTools (25), ggplot2 (26), reshape2 (27), seqinr (28), plyr (29), GO.db (30), (24)r (31), ggrepel (32), ggpointdensity (33), extrafont (34), scales (35), ggseqlogo (36), viridislite (37), xml2 (38), magrittr (39), dplyr (40), readxl (41), ggnetwork (42), STRINGdb (23, 24) and igraph (43). All data analysis scripts and annotated spectra (generated using (44)) are available at Zenodo (45) under https://doi.org/10.5281/zenodo.10581948. The R library version numbers and the R session information is stored in the file

“Text_File_S1_Session_Info_Phospho-TMT.txt” that can be accessed under the same URL. The script files for the analysis of the phosphorylation sites higher abundant under ATR inhibitor treatment can be downloaded from Zenodo via https://doi.org/10.5281/zenodo.10850046.

Extracted ion chromatogram (XIC) analysis

Protein extraction from SDS-PAGE bands, digestion with Trypsin/LysC followed the protocol described in (46), deviating in the usage of 5% hydroxylamine to stop the TMT labelling reaction. The samples were analysed on an Orbitrap Lumos (Thermo Fisher), connected to a Thermo Dionex UltiMate 3000RLSC system. The identical MS instrument settings and LC gradient as detailed in (6) were applied. Mass spectrometry raw data was searched using MaxQuant (Version 2.1.3.0) using the same FASTA file as described under “*Data analysis global phosphoproteomics*”. Standard MaxQuant search settings were applied with the additional variable modification of phosphorylation of serine, threonine and tyrosine. Peptide and protein FDR threshold was set to 5%. The TMT intensity data for the LUZP1 phosphopeptide AIGALASpSR (Spectrum #17044 in file LUM190801-16_FW052_TL-01_FW.raw) had to be extracted manually using Xcalibur QualBrowser (Version 2.2 SP1.48, Thermo Fisher) as MaxQuant did not detect these signals although they are clearly present in the raw data. Data were analysed using modified versions of the R scripts used in (6, 46). In summary, data were normalized using VSN and the TMT reporter intensities were statistically tested using t-tests under application of Bonferroni correction of the significance threshold to $\alpha = 0.05 / 3 = 0.0167$ (3 t-tests). All mass spectrometry raw data, MaxQuant search settings and output files were uploaded to jPOSTrepo and can be downloaded via ProteomeXchange with the identifier PXD040476. Data analysis scripts and annotated spectra (44) can be downloaded from Zenodo under https://doi.org/10.5281/zenodo.10581706.

Samples from DHX9 immunoprecipitation were analysed after SDS-PAGE, digestion and peptide extraction (see main manuscript text) as follows: Peptides were resuspended in 5%

formic acid in water and injected on an UltiMate 3000 RSLCnano System coupled to an Orbitrap Exploris 240 (Thermo Scientific). Peptides were loaded on an Acclaim PepMap trap column (Thermo Scientific #164750) prior analysis on a PepMap RSLC C18 analytical column (Thermo Scientific #ES903) and eluted on a 60 min linear gradient from 3 to 35% Buffer B (Buffer A: 0.1% formic acid in water, Buffer B: 0.08% formic acid in 80:20 acetonitrile:water (v:v)). Eluted peptides were then analysed by the mass spectrometer operating in data dependent acquisition mode using a cycle time of 2 s. MS1 were acquired at a resolution of 60000 with an AGC target of 300% and a maximum injection time of 25 ms. Peptides were then selected for HCD fragmentation using an isolation width of 1.2 Th, NCE of 30%, AGC of 100% and maximum injection time of 100 ms and MS2 were acquired at a resolution of 15000. Dynamic exclusion was set to 30 s with a tolerance of +/- 10 ppm. Mass spectrometry raw data was searched using MaxQuant (Version 2.2.0.0) against a Uniprot *homo sapiens* FASTA (42,397 entries inclusive isoforms, downloaded 2nd January 2023 from www.uniprot.org). In addition to the standard MaxQuant search parameter, deamidation of N and Q, and phosphorylation of S, T and Y was added as variable modifications. Intensities of peptides from DHX9 which were repeatedly measured were averaged using their median intensity and then all DHX9 peptides from the different samples were normalized via VSN. Data was then statistically tested using limma. The mass spectrometry raw data, MaxQuant search settings and output files were uploaded to PRIDE (45) and can be accessed via ProteomeXchange (PXD041250). Data analysis scripts and annotated spectra (44) were uploaded to Zenodo and can be downloaded via https://doi.org/10.5281/zenodo.10882997. In case of DHX9 from HeLa and HEK293 cells, the immunoprecipitated samples were separated via SDS-PAGE, the relevant protein bands were cut-out and digested and peptides extracted as described in the main manuscript text. Afterwards, the peptides were resuspended in 0.1% (v/v) formic acid and 0.015% (w/v) n-Dodecyl-beta-maltoside. Samples were analysed using an UltiMate 3000 RSLCnano System coupled to an Orbitrap Exploris 240 (Thermo Fisher Scientific). Peptides were trapped on an

Acclaim PepMap trap column (Thermo Fisher Scientific #164750) and separated on a PepMap RSLC C18 analytical column (Thermo Fisher Scientific #ES903). Peptides were eluted using a 60 min linear gradient from 3 to 35% Buffer B (Buffer A: 0.1% (v/v) formic acid in H_2O, Buffer B: 0.08% (v/v) formic acid in 80:20 acetonitrile:H_2O (v:v)) and analysed using parallel reaction monitoring mass spectrometry. In brief, the mass spectrometer conducted two consecutive MS1 scans spanning a width of 2 Da, centring at 538.2802 m/z or 578.2633 m/z, respectively (60,000 resolution). These two masses correspond to the non-phosphorylated and phosphorylated forms of the doubly charged peptide LAQFEP**S**QR (Ser321 of DHX9). This was followed by two MS/MS scans using the same MS1 mass windows at a resolution of 15,000 with a normalised collision energy of 30. Automatic gain control was set to “Standard” and maximum injection time mode was set to “Auto”. As first step the elution time window of the phospho-peptide was established. Fort this, the raw data was searched using MaxQuant (version 2.2.0.0) as stated above, with variable modifications set to oxidation of methionine, acetylation of protein N-terminus and phosphorylation of serine and threonine. Raw data was then converted to mzML using the MSConvertGUI tool of ProteoWizard (version 3.0.23301-4fd35e2) (46), enabling peak picking (Vendor algorithm) on MS levels 1-2 and enabling the “SIM as spectra” option. Data was then analysed in R (version 4.3.1) for the coincidence of the precursor mass (578.2633 *m/z*) and the highest abundant fragment ion of the phospho-peptide (neutral loss of y4 (y4*): 469.2518 *m/z*) (see representative spectrum in Figure S9). This coincidence appeared with increased density during an elution time window which overlapped with the elution time window as established by the MaxQuant analysis. By this the elution time window of the phospho-peptide in all samples was established for subsequent analysis in Skyline (v23.1.0.380 (cf25ad847)) (47, 48) monitoring b1-8 and y1-8 ions and manual integration of the corresponding peaks. The Skyline transition data was exported as comma separated values. This file was processed in R (version 4.3.1), excluding the fragment ions y1-3 and b1-3 of both the phospho and non-phosphopeptide. First, the respective background area was subtracted from the area of each

individual detected fragment ion. The background corrected areas were then summed up and standardized by dividing the area of the phospho-peptide by the sum of the area of the non-phosphopeptide and phospho-peptide. This ratio was then $\log_2$ transformed and statistically tested using a two-tailed student's t-test under assumption of equal variance between the two tested groups. A p-value ≤ 0.05 was regarded as evidence for a difference in the mean between the tested groups. The analysis scripts for the elution time window establishment of the phospho-peptide, the Skyline analysis raw data and all annotated spectra can be downloaded from Zenodo via https://doi.org/10.5281/zenodo.10882997. The raw files and MaxQuant search results are available under jPOSTrepo (HeLa: PXD050953, HEK-293: PXD050954).

SUPPLEMENTARY FIGURES AND FIGURE LEGENDS

Figure S1

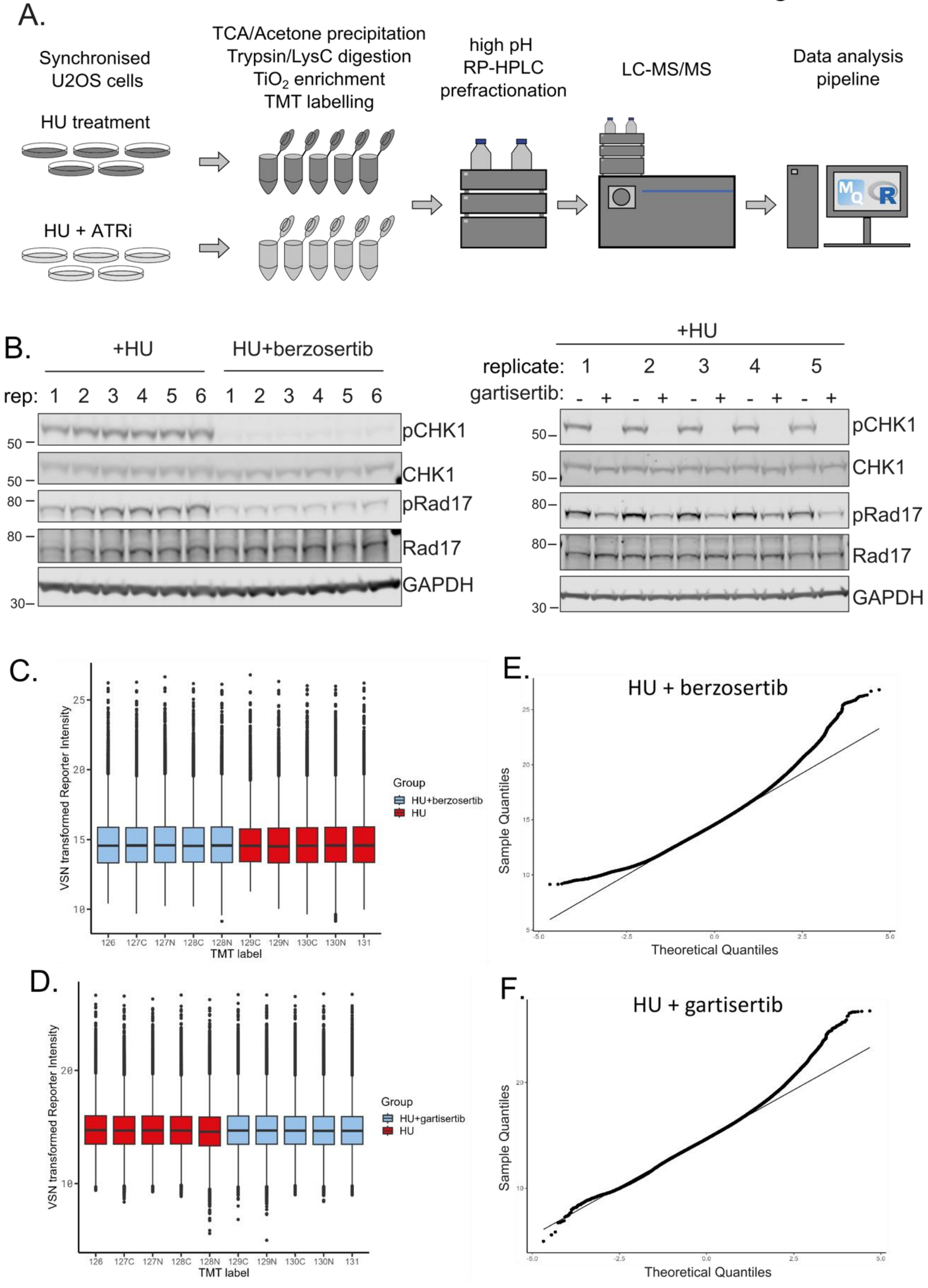
A.
Synchronised U2OS cells
HU treatment
HU + ATRi
TCA/Acetone precipitation
Trypsin/LysC digestion
TiO2 enrichment
TMT labelling
high pH RP-HPLC prefractionation
LC-MS/MS
Data analysis pipeline
B.
+HU
HU+berzosertib
rep: 1 2 3 4 5 6 1 2 3 4 5 6
pCHK1
CHK1
pRad17
Rad17
GAPDH
+HU
replicate: 1 2 3 4 5
gartisertib: - + - + - + - + - +
C.
VSN transformed Reporter Intensity
TMT label
Group
HU+berzosertib
HU
D.
VSN transformed Reporter Intensity
TMT label
Group
HU+gartisertib
HU
E.
HU + berzosertib
Sample Quantiles
Theoretical Quantiles
F.
HU + gartisertib
Sample Quantiles
Theoretical Quantiles

Figure S1. Quantitative phosphoproteomic screening workflow. (A). Quantitative phosphoproteomic screening workflow. (B). Assessment of ATR activity in the 5 biological replicates used in the HU±berzosertib and HU±gartisertib phosphoproteomic screens. U-2OS cells synchronized in S-phase (11h after release from nocodazole) were pre-incubated for 1h, or not, with berzosertib or gartisertib (1 µM) before addition of HU (0.5 mM) for 0.5h. Cell extracts were subjected to western blotting with the antibodies indicated. (C and D). Boxplot of intensity distribution in each TMT channel, HU±berzosertib screen (C); HU±gartisertib screen (D). No obvious discrepancy between the median values of the individual channels indicates a successful calibration by VSN and no introduction of an obvious intensity bias for any experimental group. (E and F). Normal QQ-Plots of the TMT intensity data from the HU±berzosertib screen (E) and HU±gartisertib screen (F) after VSN transformation. Only minor deviations from the line indicates the transformed data follows a normal distribution to a satisfactory degree. The hypervariable datapoints in the upper quantiles are controlled by the application of the robust implementation of the empirical Bayes algorithm used by limma (9) and implemented in the analysis scripts.

Figure S2

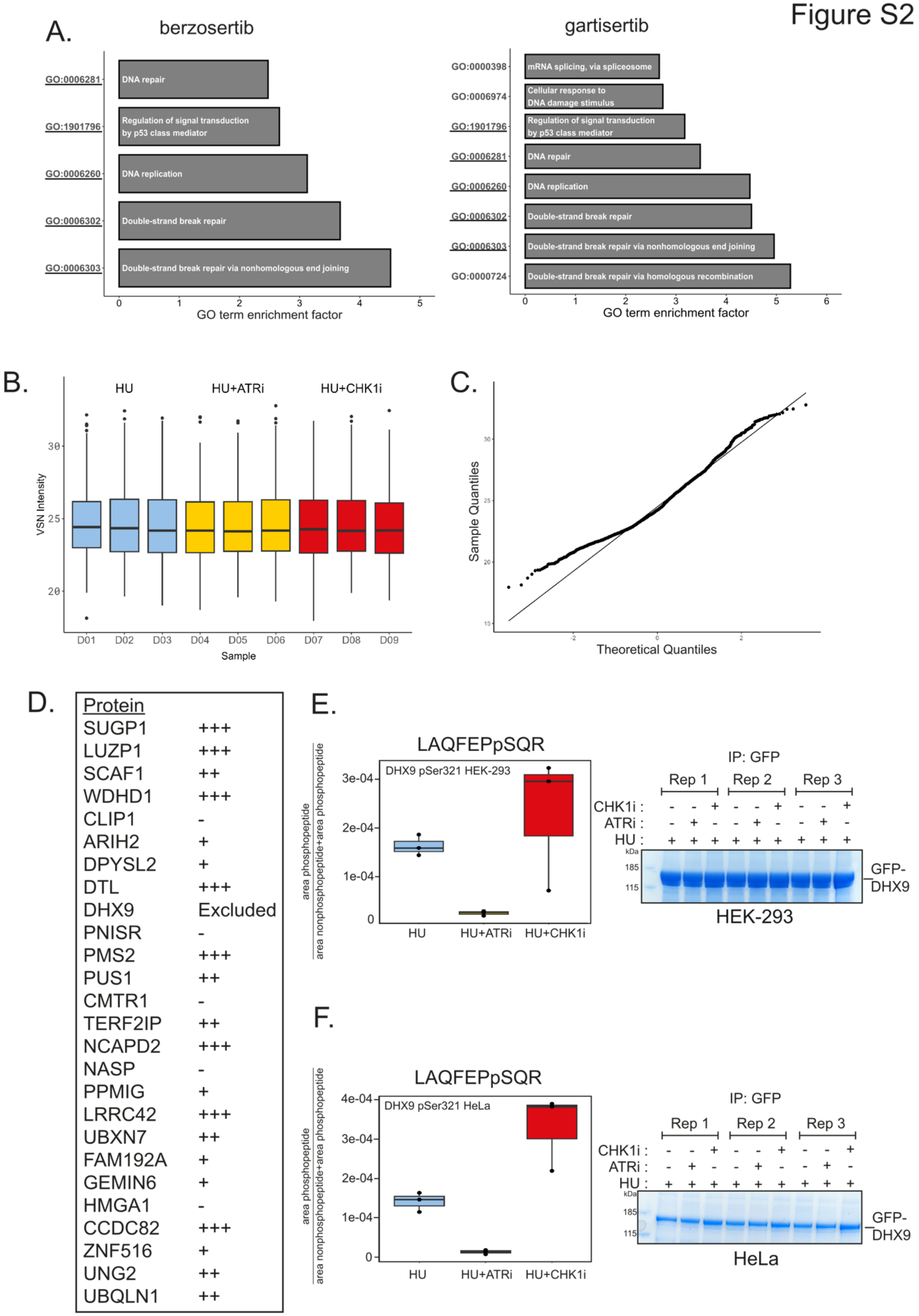
A.
berzosertib
GO:0006281 DNA repair
GO:1901796 Regulation of signal transduction by p53 class mediator
GO:0006260 DNA replication
GO:0006302 Double-strand break repair
GO:0006303 Double-strand break repair via nonhomologous end joining
GO term enrichment factor
gartisertib
GO:0000398 mRNA splicing, via spliceosome
GO:0006974 Cellular response to DNA damage stimulus
GO:1901796 Regulation of signal transduction by p53 class mediator
GO:0006281 DNA repair
GO:0006260 DNA replication
GO:0006302 Double-strand break repair
GO:0006303 Double-strand break repair via nonhomologous end joining
GO:0000724 Double-strand break repair via homologous recombination
GO term enrichment factor
B.
HU
HU+ATRi
HU+CHK1i
VSN Intensity
D01 D02 D03 D04 D05 D06 D07 D08 D09
Sample
C.
Sample Quantiles
Theoretical Quantiles
D.
Protein
SUGP1 +++
LUZP1 +++
SCAF1 ++
WDHD1 +++
CLIP1 -
ARIH2 +
DPYSL2 +
DTL +++
DHX9 Excluded
PNISR -
PMS2 +++
PUS1 ++
CMTR1 -
TERF2IP ++
NCAPD2 +++
NASP -
PPMIG +
LRRC42 +++
UBXN7 ++
FAM192A +
GEMIN6 +
HMGA1 -
CCDC82 +++
ZNF516 +
UNG2 ++
UBQLN1 ++
E.
LAQFEPpSQR
DHX9 pSer321 HEK-293
area phosphopeptide / area nonphosphopeptide+area phosphopeptide
HU HU+ATRi HU+CHK1i
IP: GFP
Rep 1 Rep 2 Rep 3
CHK1i :
ATRi :
HU :
kDa
185
115
GFP-DHX9
HEK-293
F.
LAQFEPpSQR
DHX9 pSer321 HeLa
area phosphopeptide / area nonphosphopeptide+area phosphopeptide
HU HU+ATRi HU+CHK1i
IP: GFP
Rep 1 Rep 2 Rep 3
CHK1i :
ATRi :
HU :
kDa
185
115
GFP-DHX9
HeLa

Figure S2. Analysis of Gene Ontology and protein recruitment. (A). Gene Ontology terms enriched among proteins whose phosphorylation was inhibited by berzosertib or gartisertib. Significance cut–off was set as $\alpha = 0.01$ with at least 2 proteins identified in the respective group. (B). Boxplots of the VSN-adjusted ion intensities for all peptides for each condition in the case of GFP-DHX9 from the experiment in (Fig 3C). (C). Normal QQ-Plots of the peptide intensity data from the experiment in (Fig 3C) after VSN transformation. (D). Relative strength of protein recruitment to DNA damage sites in laser micro-irradiation experiments. (E). Boxplot of the abundance of the peptide LAQFEPpSQR (pS321) of DHX9 under HU stress (HU), HU + ATR inhibitor treatment (HU+ATRi) and HU + CHK1 inhibitor treatment (HU+CHK1i) as measured by parallel reaction monitoring (PRM). Change in phosphopeptide abundance as derived from HEK-293 cells (left panel, fold-change HU vs. HU+ATRi: -6.33; $p = 0.00017$), right panel shows the GFP-IP after SDS PAGE and Coomassie staining. Bands corresponding to GFP-DHX9 were excised and used for MS analysis (Rep = biological Replicate). (F). Boxplots for DHX9 pS321 from HeLa cells (left panel, fold-change HU vs. HU+ATRi: -10.46; $p = 0.0010$). Right panel shows the GFP-IP after SDS PAGE and Coomassie staining. Bands corresponding to GFP-DHX9 were excised and used for MS analysis (Rep = biological Replicate). Raw mass spectrometry files are available from jPOSTrepo (HeLa: PXD050953, HEK293: PXD050954), annotated spectra and Skyline analysis results from Zenodo under https://doi.org/10.5281/zenodo.10882997.

Figure S3

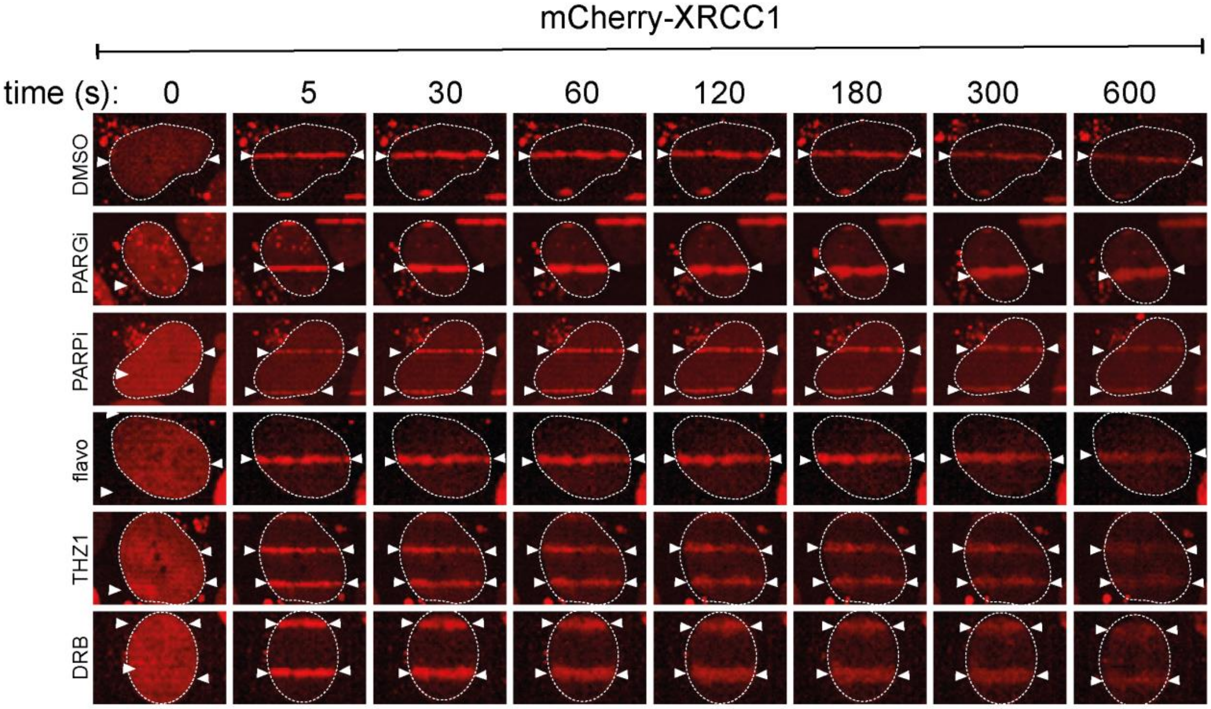

Figure S3. Transcriptional inhibitors do not affect XRCC1 recruitment to DNA damage sites. BrdU–sensitized U–2 OS Flp–In T–REx cells stably expressing mCherry-tagged XRCC1, pre–incubated with DMSO, olaparib (5 µM; PARPi), PDD00017273 (0.3 µM; PARGi), flavopiridol (10 µM), THZ-1 (10 µM) or DRB for 1 h were micro-irradiated with a 405 nm laser and imaged at the times indicated.

Figure S4

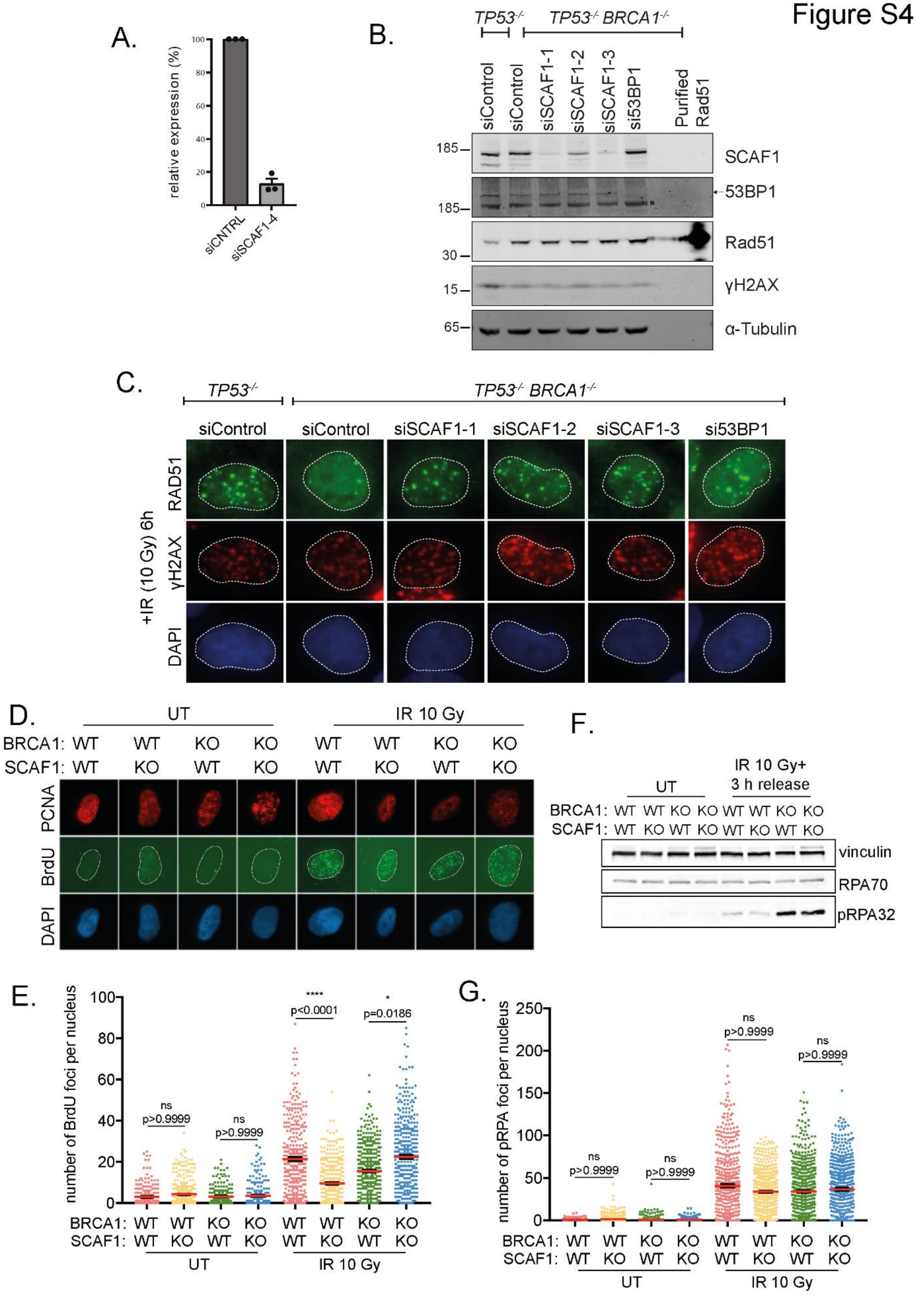
A.
relative expression (%)
siCNTRL
siSCAF1-4
B.
TP53-/-
TP53-/- BRCA1-/-
siControl
siControl
siSCAF1-1
siSCAF1-2
siSCAF1-3
si53BP1
Purified Rad51
SCAF1
53BP1
Rad51
γH2AX
α-Tubulin
C.
TP53-/-
TP53-/- BRCA1-/-
siControl
siControl
siSCAF1-1
siSCAF1-2
siSCAF1-3
si53BP1
+IR (10 Gy) 6h
RAD51
γH2AX
DAPI
D.
UT
IR 10 Gy
BRCA1: WT WT KO KO WT WT KO KO
SCAF1: WT KO WT KO WT KO WT KO
PCNA
BrdU
DAPI
F.
UT
IR 10 Gy+ 3 h release
BRCA1: WT WT KO KO WT WT KO KO
SCAF1: WT KO WT KO WT KO WT KO
vinculin
RPA70
pRPA32
E.
number of BrdU foci per nucleus
ns p>0.9999
ns p>0.9999
**** p<0.0001
* p=0.0186
BRCA1: WT WT KO KO WT WT KO KO
SCAF1: WT KO WT KO WT KO WT KO
UT
IR 10 Gy
G.
number of pRPA foci per nucleus
ns p>0.9999
ns p>0.9999
ns p>0.9999
ns p>0.9999
BRCA1: WT WT KO KO WT WT KO KO
SCAF1: WT KO WT KO WT KO WT KO
UT
IR 10 Gy

Figure S4. SCAF1 depletion restores RAD1 foci in BRCA1-knockout cells. (A). qPCR analysis of SCAF1 knock-down upon siRNA transfection of RPE1 hTERT *TP53*$^{-/-}$ *BRCA1*$^{-/-}$ cells with siSCAF1-4 (SN lab). SCAF1 expression was normalized to expression of the house keeping gene GUSB and normalized expression of SCAF1 in siCNTRL samples was set at 100% per biological replicate (n=3). (B). The cell lines shown were transfected with the siRNAs indicated. Cells were lysed and extracts subjected to western blotting with the antibodies indicated. (C). The cell lines indicated were transfected with the siRNAs indicated and after 24h cells were exposed to IR (10 Gy). Cells were allowed to recover for 3h, fixed and subjected to immunofluorescence to detect RAD51 foci. (D). Representative images of immunofluorescence staining against BrdU and PCNA in RPE1 hTERT *TP53*$^{-/-}$ that are wild type (WT) or knockout (KO) for BRCA1 and/or SCAF1 as indicated. (E). Quantification of the number of BrdU foci in S-phase RPE1 hTERT *TP53*$^{-/-}$ cells with the *BRCA1* and *SCAF1* genotypes indicated: WT, wildtype, KO, knockout determined by PCNA staining before and after exposure of cells to ionising radiation (IR; 10 Gy). Data is shown with mean ± SEM from 3 independent experiments. $^{*}p<0.1$ and $^{****}p < 0.0001$ (one-way ANOVA, followed by Kruskal-Wallis test). (F). Levels of RPA70 and phospho-RPA32 (pS4+pS8) in RPE1 hTERT *TP53*$^{-/-}$ cells of the genotypes indicated treated with or without 10 Gy ionising radiation (IR). Antibodies against vinculin were used as a loading control. (G). Quantification of the number of phospho-RPA32 (pS4+pS8) foci in S-phase RPE1 hTERT *TP53*$^{-/-}$ cells with the *BRCA1* and *SCAF1* genotypes indicated, determined by PCNA staining before and after exposure of cells to ionising radiation (IR; 10 Gy). Data is shown with mean ± SEM from 3 independent experiments. ns: p>0.9999 (One-way ANOVA, followed by Kruskal-Wallis test). UT, untreated.

Figure S5

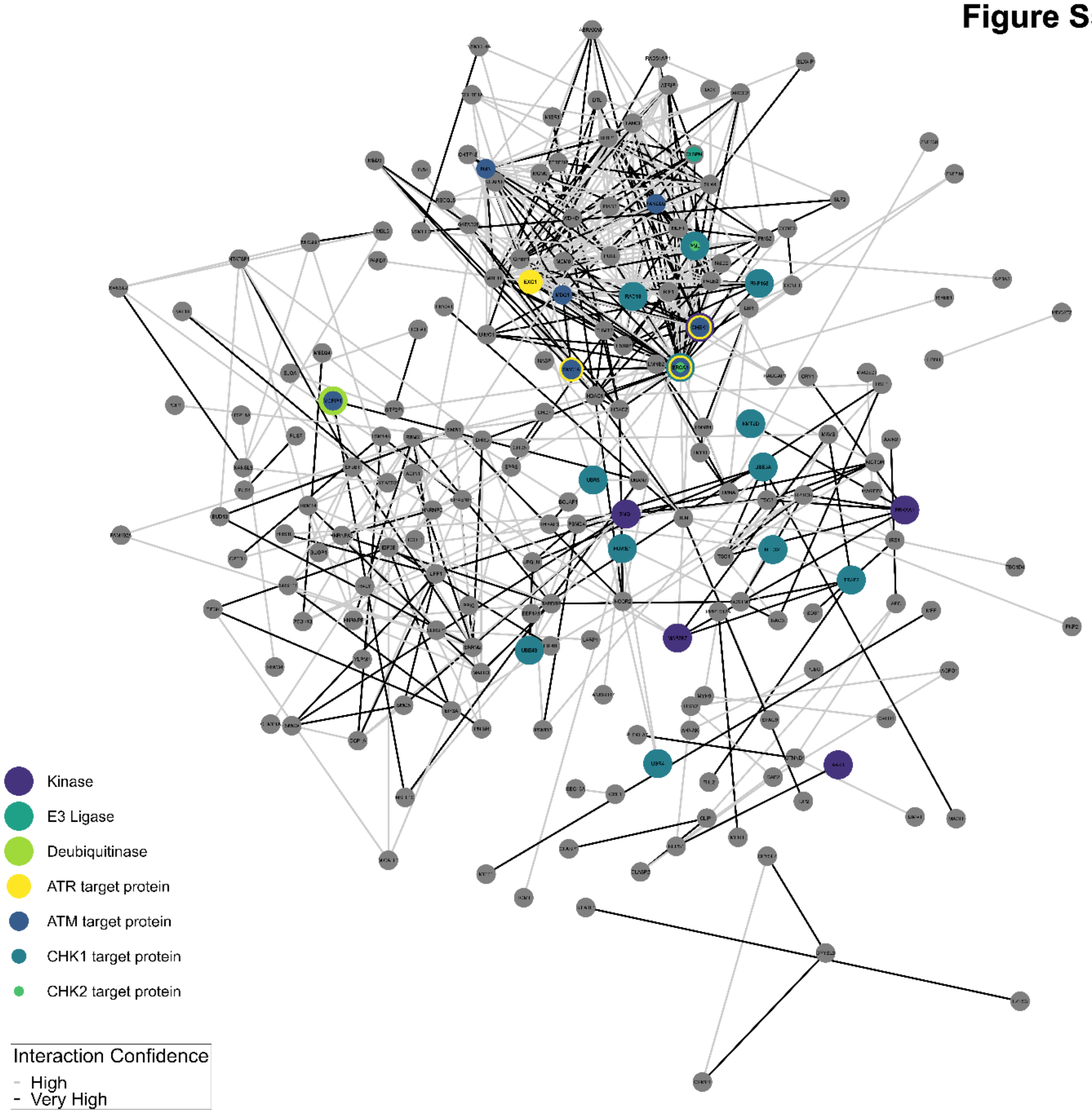

Figure S5. Protein interaction network relating to phosphopeptides downregulated after gartisertib. Analysis was performed using STRINGdb (23, 42) using R script files available from https://doi.org/10.5281/zenodo.10581948. Interaction confidence scores (High: ≥ 700 (light grey edges); Very High ≥ 900 (dark grey edges)) provided by STRINGdb.

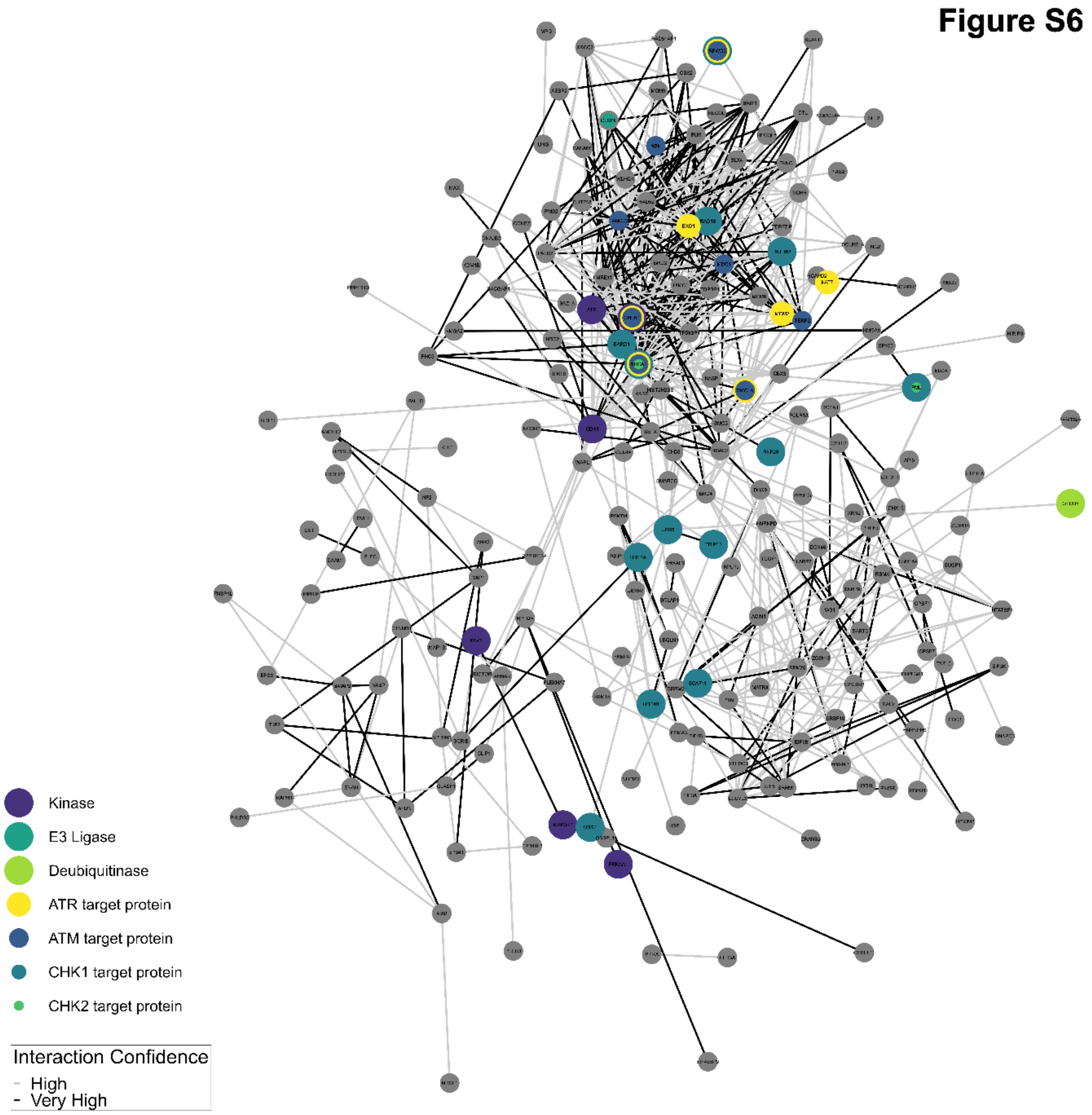

Figure S6. Protein interaction network relating to phosphopeptides downregulated after berzosertib. Analysis was performed using STRINGdb (23, 24) using R script files available from https://doi.org/10.5281/zenodo.10581948. Interaction confidence scores (High: ≥ 700 (light grey edges); Very High ≥ 900 (dark grey edges)) provided by STRINGdb.

Figure S7

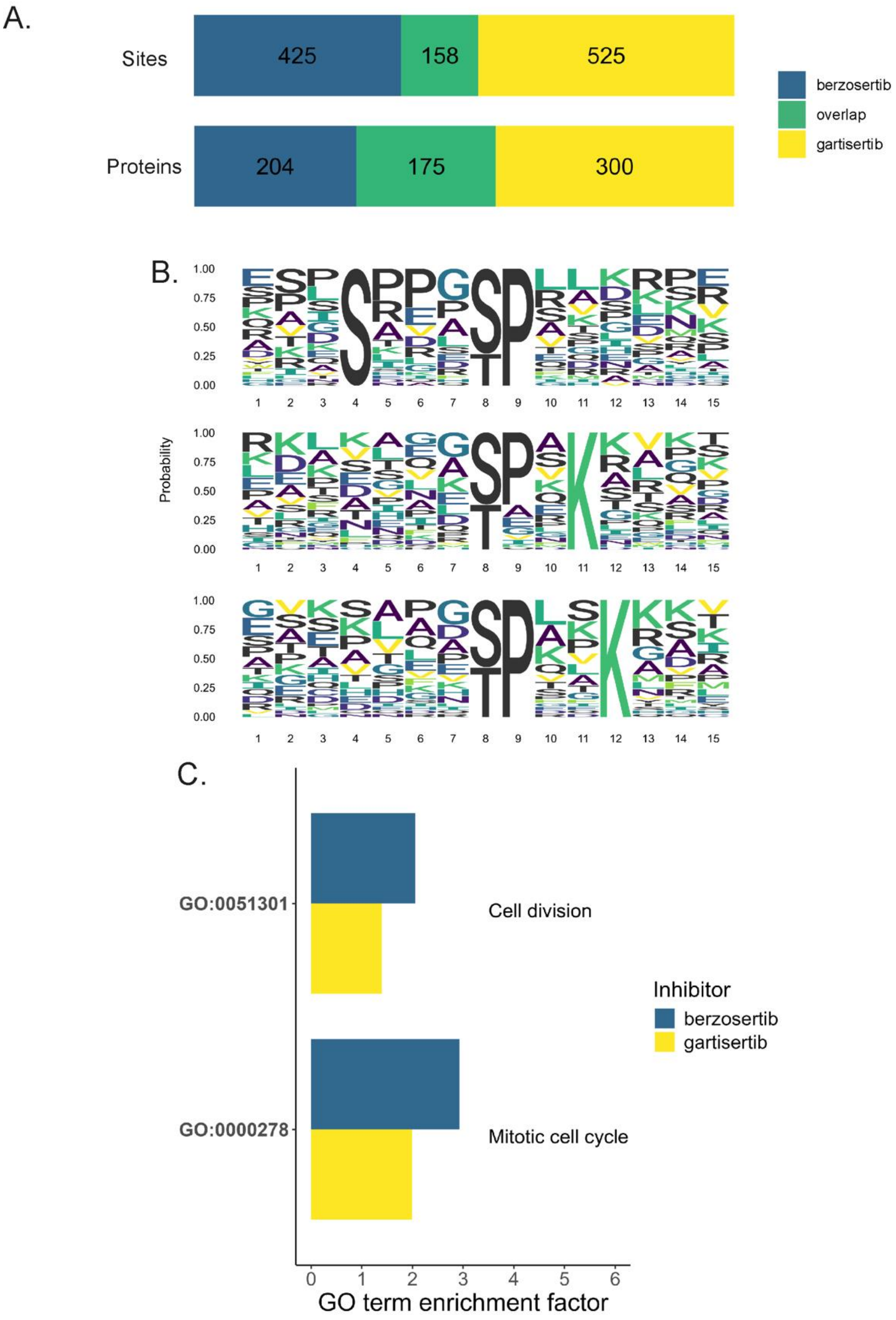
A.
Sites
425
158
525
Proteins
204
175
300
berzosertib
overlap
gartisertib
B.
Probability
1.00
0.75
0.50
0.25
0.00
1 2 3 4 5 6 7 8 9 10 11 12 13 14 15
C.
GO:0051301
Cell division
GO:0000278
Mitotic cell cycle
Inhibitor
berzosertib
gartisertib
0 1 2 3 4 5 6
GO term enrichment factor

Figure S7. Proteins with phosphorylation sites upregulated upon ATR inhibition.

A. Phosphorylation sites as called by MaxQuant and is here not FLR controlled. B. Sequence motifs detected in higher abundant phosphopeptides occurring common under both ATR inhibitor treatment regimes. Only peptides with single phosphorylation sites exhibiting a ≤ 1% FLR were taken into account.C. Gene Ontology terms of proteins with higher abundant phosphorylation sites common to gartisertib and berzosertib. The enrichment factor is calculated as the ratio of the portion of GO term occurrences within the set of proteins with higher abundant phosphorylation sites under inhibitor treatment and the portion of the GO term occurring in the complete protein set.

Figure S8

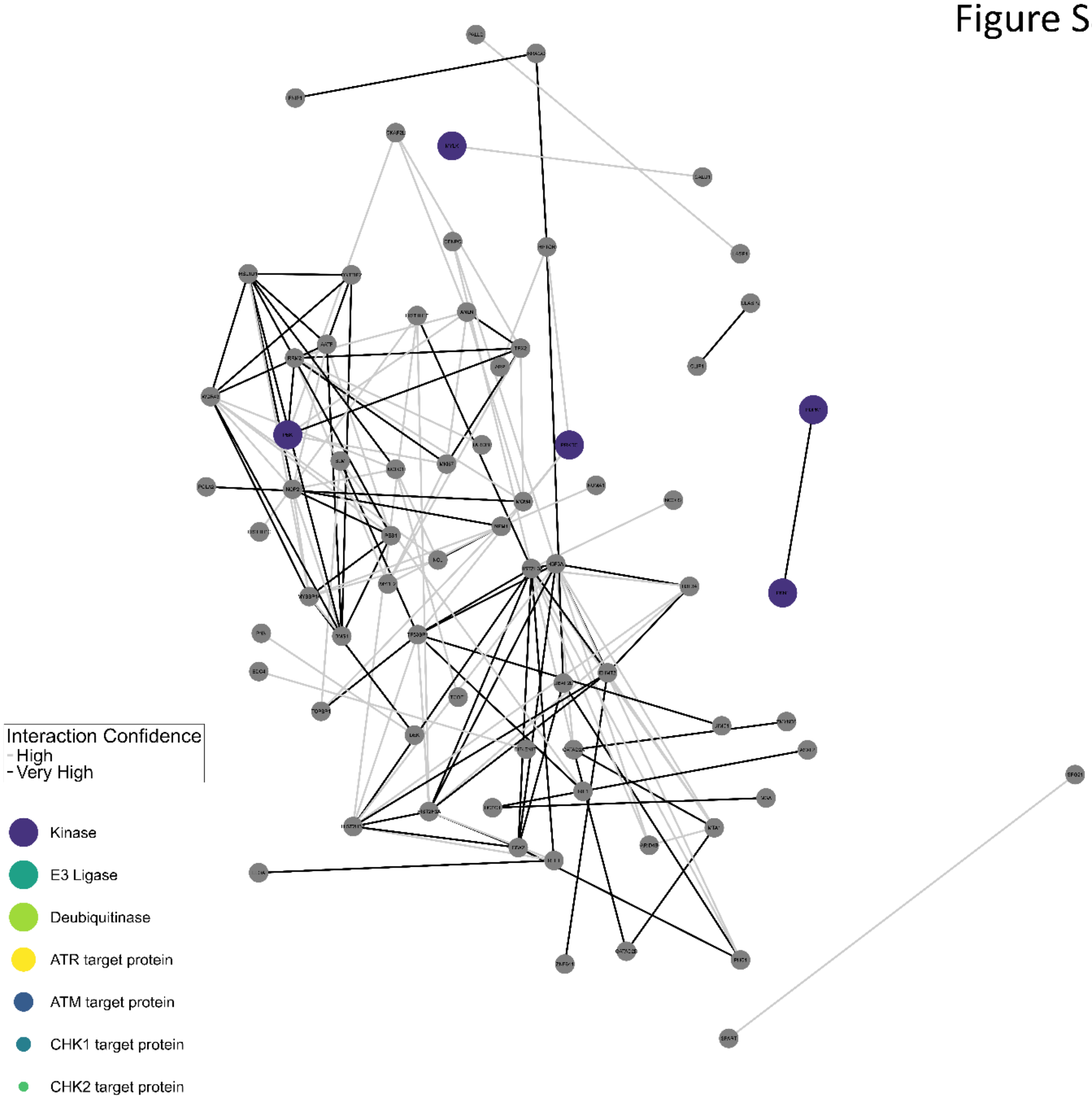

Fig. S8 Protein interaction network relating to phosphopeptides commonly upregulated after gartisertib & berzosertib. Analysis was performed using STRINGdb (23, 24) using R script files available from https://doi.org/10.5281/zenodo.10850046. Interaction confidence scores (High: ≥ 700 (light grey edges); Very High ≥ 900 (dark grey edges)) provided by STRINGdb.

Figure S9

Representative annotated spectrum of the peptide LAQFEPpSQR (pSer321) of DHX9 as identified by MaxQuant

Sample C2 (HU + Chk1 inhibitor treatment)

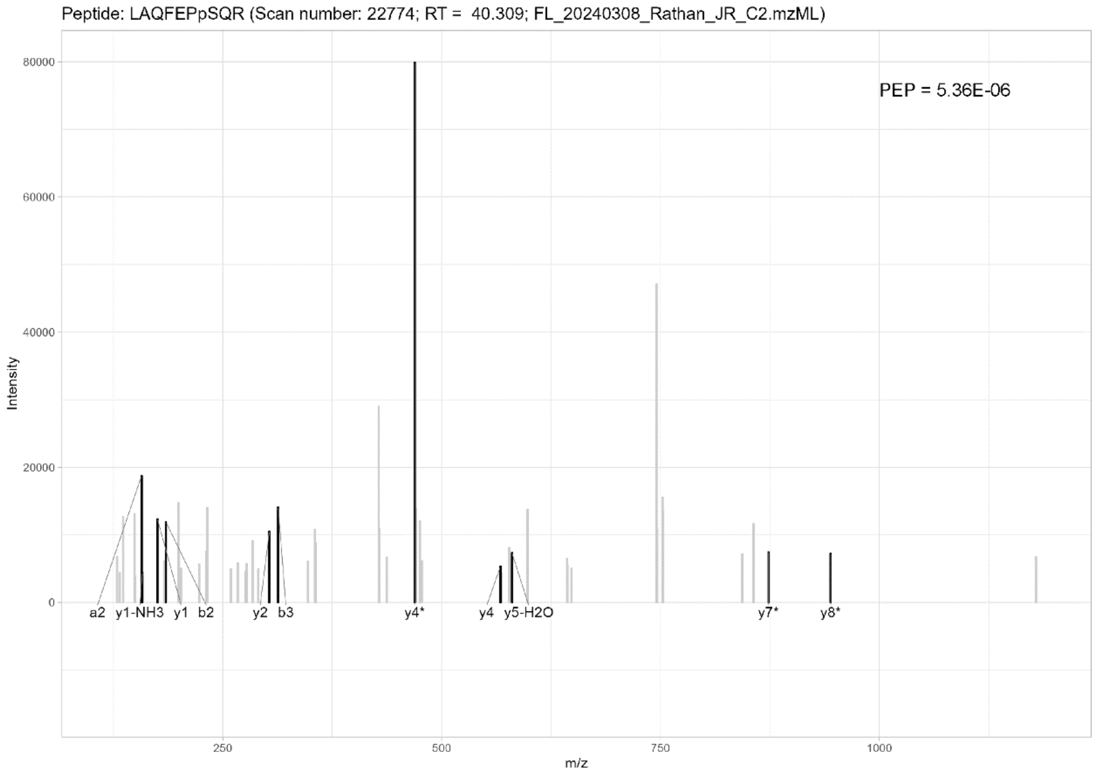

Figure S9. Representative annotated spectrum of the DHX9 Ser321 phosphopeptide LAQFEPpSQR. Bars in black indicate fragment ion masses as per MaxQuant analysis, bars in grey represent non-matched masses. RT: Retention time in minutes; PEP: Posterior error probability of peptide identification. All annotated spectra are available via Zenodo: https://doi.org/10.5281/zenodo.10882997.

Supplementary Tables

Table S1. Mass spectrometry summary statistics. Numbers of identified and quantified peptide to spectrum matches (PSM), (phospho)peptides in the phosphopeptide-enriched samples from U-2 OS cells ± berzosertib or gartisertib.

Table S2. Phosphoproteomic dataset: U-2 OS cells, HU ± berzosertib. Quantitative proteomics dataset showing all phosphopeptides identified in U-2 OS cells treated with HU ± berzosertib. Phosphopeptides with identical sequence and post–translational modifications which were detected in the same chromatography fraction, had their respective TMT channel intensities averaged and given unique identifier numbers (column A "peptide number"). A given peptide with a unique identifier number can appear multiple times in the table if there is more than a single phosphorylation site and/or MaxQuant assigns a set of possible phosphorylation site localisation probabilities for a site (values listed under "PTM score probability", column I). For statistical analysis, each peptide with a unique identifier number was considered exactly once, independent of the number of entries in this table. A phosphorylation localisation site probability threshold of 0.994 was applied to give an FLR cut-off of 1% (11). The mass spectrometry phosphoproteomics data have been deposited to the ProteomeXchange Consortium via jPOSTrepo (PXD040469).

Table S3. Phosphoproteomic dataset: U-2 OS cells, HU ± gartisertib. Quantitative proteomics dataset of all showing all phosphopeptides identified in U-2 OS cells treated with HU ± gartisertib. Phosphopeptides with identical sequence and post–translational modifications which were detected in the same chromatography fraction, had their respective TMT channel intensities averaged and given unique identifier numbers (column A "peptide number"). A given peptide with a unique identifier number can appear multiple times in the table if there is more than a single phosphorylation site and/or MaxQuant assigns a range of possible phosphorylation site localisation probabilities for a site (values listed under "PTM score

probability”, column I). For statistical analysis, each peptide with a unique identifier number was considered exactly once, independent of the number of entries in this table. A phosphorylation localisation site probability threshold of 0.994 was applied to give an FLR cut-off of 1% (11). The mass spectrometry phospho-proteomics data have been deposited to the ProteomeXchange Consortium via jPOSTrepo (PXD040469).

Table S4. Overlap between hits in Table S2 (HU ± berzosertib) and Table S3 (HU ± gartisertib). Phosphorylation sites downregulated by both gartisertib and berzosertib (independent of the phosphopeptide detected). Phosphorylated amino acid is shown surrounded by square brackets in column K (“Motif”).

Table S5. New ATR targets already implicated in cell responses to DNA damage or replication stress. Phospho-sites down-regulated by berzosertib and gartisertib. Phosphosites appearing in different peptides are marked in grey. Phosphorylated amino acid is indicated in red in column K (“Motif”).

Table S6. New ATR targets not previously implicated in cell responses to DNA damage or replication stress. Phospho-sites down-regulated by berzosertib and gartisertib. Phosphosites appearing in different peptides are marked in grey. The phosphorylated amino acid is preceded by a “p” in column F (“Peptide sequence”).

Table S7. Phosphorylation sites upregulated by gartisertib and berzosertib. 5% FDR cut-off for differential abundance between HU treatment and HU + ATRi. The phosphorylation site is assigned as per MaxQuant analysis and is here not FLR controlled.

Table S8. Proteins with Phosphorylation sites upregulated by gartisertib and berzosertib. 5% FDR cut-off for differential abundance between HU treatment and HU + ATRi. Proteins in this table do not need to contain the same higher abundant phosphorylation site to be included.

Table S9. Lists of the reagents, antibodies, plasmid constructs, siRNA sequences, peptide sequences and primer sequences used in this study. Datasheets for each plasmid used in this study will be available on a dedicated page of our Reagents website upon publication.

SUPPLEMENTARY REFERENCES

1. UniProt, C. (2021) UniProt: the universal protein knowledgebase in 2021. *Nucleic Acids Res* 49, D480-D489
2. Tyanova, S., Temu, T., and Cox, J. (2016) The MaxQuant computational platform for mass spectrometry-based shotgun proteomics. *Nat Protoc* 11, 2301-2319
3. Okuda, S., Watanabe, Y., Moriya, Y., Kawano, S., Yamamoto, T., Matsumoto, M., Takami, T., Kobayashi, D., Araki, N., Yoshizawa, A. C., Tabata, T., Sugiyama, N., Goto, S., and Ishihama, Y. (2017) jPOSTrepo: an international standard data repository for proteomes. *Nucleic Acids Res* 45, D1107-D1111
4. Deutsch, E. W., Bandeira, N., Perez-Riverol, Y., Sharma, V., Carver, J. J., Mendoza, L., Kundu, D. J., Wang, S., Bandla, C., Kamatchinathan, S., Hewapathirana, S., Pullman, B. S., Wertz, J., Sun, Z., Kawano, S., Okuda, S., Watanabe, Y., MacLean, B., MacCoss, M. J., Zhu, Y., Ishihama, Y., and Vizcaino, J. A. (2023) The ProteomeXchange consortium at 10 years: 2023 update. *Nucleic Acids Res* 51, D1539-D1548
5. R.Core.Team (2021) *R: A Language and Environment for Statistical Computing*, R Foundation for Statistical Computing, Vienna, Austria
6. Khanam, T., Muñoz, I., Weiland, F., Carroll, T., Morgan, M., Borsos, B. N., Pantazi, V., Slean, M., Novak, M., Toth, R., Appleton, P., Pankotai, T., Zhou, H., and Rouse, J. (2021) CDKL5 kinase controls transcription-coupled responses to DNA damage. *EMBO J* 40, e108271
7. Huber, W., von Heydebreck, A., Sültmann, H., Poustka, A., and Vingron, M. (2002) Variance stabilization applied to microarray data calibration and to the quantification of differential expression. *Bioinformatics* 18 Suppl 1, S96-104
8. Huber, W., von Heydebreck, A., Sueltmann, H., Poustka, A., and Vingron, M. (2003) Parameter estimation for the calibration and variance stabilization of microarray data. *Stat Appl Genet Mol Biol* 2, Article3
9. Phipson, B., Lee, S., Majewski, I. J., Alexander, W. S., and Smyth, G. K. (2016) ROBUST HYPERPARAMETER ESTIMATION PROTECTS AGAINST HYPERVARIABLE GENES AND IMPROVES POWER TO DETECT DIFFERENTIAL EXPRESSION. *Ann Appl Stat* 10, 946-963
10. Ritchie, M. E., Phipson, B., Wu, D., Hu, Y., Law, C. W., Shi, W., and Smyth, G. K. (2015) limma powers differential expression analyses for RNA-sequencing and microarray studies. *Nucleic Acids Res* 43, e47
11. Ferries, S., Perkins, S., Brownridge, P. J., Campbell, A., Eyers, P. A., Jones, A. R., and Eyers, C. E. (2017) Evaluation of Parameters for Confident Phosphorylation Site Localization Using an Orbitrap Fusion Tribrid Mass Spectrometer. *J Proteome Res* 16, 3448-3459
12. Wagih, O., Sugiyama, N., Ishihama, Y., and Beltrao, P. (2016) Uncovering Phosphorylation-Based Specificities through Functional Interaction Networks. *Mol Cell Proteomics* 15, 236-245
13. Storey, J. D., Bass, A. J., Dabney, A., and Robinson, D. (2022) *qvalue: Q-value estimation for false discovery rate control*, http://github.com/jdstorey/qvalue
14. Stark, C., Breitkreutz, B.-J., Reguly, T., Boucher, L., Breitkreutz, A., and Tyers, M. (2006) BioGRID: a general repository for interaction datasets. *Nucleic Acids Res* 34, D535-539
15. Hornbeck, P. V., Zhang, B., Murray, B., Kornhauser, J. M., Latham, V., and Skrzypek, E. (2015) PhosphoSitePlus, 2014: mutations, PTMs and recalibrations. *Nucleic Acids Res* 43, D512-520
16. Cunningham, F., Allen, J. E., Allen, J., Alvarez-Jarreta, J., Amode, M. R., Armean, I. M., Austine-Orimoloye, O., Azov, A. G., Barnes, I., Bennett, R., Berry, A., Bhai, J., Bignell, A., Billis, K., Boddu, S., Brooks, L., Charkhchi, M., Cummins, C., Da Rin Fioretto, L., Davidson, C., Dodiya, K., Donaldson, S., El Houdaigui, B., El Naboulsi, T., Fatima, R., Giron, C. G., Genez, T., Martinez, J. G., Guijarro-Clarke, C., Gymer, A., Hardy, M., Hollis, Z., Hourlier, T., Hunt, T., Juettemann, T., Kaikala, V., Kay, M., Lavidas, I., Le, T., Lemos, D., Marugán, J. C., Mohanan, S., Mushtaq, A., Naven, M., Ogeh, D. N., Parker, A., Parton, A.,

Perry, M., Piližota, I., Prosovetskaia, I., Sakthivel, M. P., Salam, A. I. A., Schmitt, B. M., Schuilenburg, H., Sheppard, D., Pérez-Silva, J. G., Stark, W., Steed, E., Sutinen, K., Sukumaran, R., Sumathipala, D., Suner, M.-M., Szpak, M., Thormann, A., Tricomi, F. F., Urbina-Gómez, D., Veidenberg, A., Walsh, T. A., Walts, B., Willhoft, N., Winterbottom, A., Wass, E., Chakiachvili, M., Flint, B., Frankish, A., Giorgetti, S., Haggerty, L., Hunt, S. E., Iisley, G. R., Loveland, J. E., Martin, F. J., Moore, B., Mudge, J. M., Muffato, M., Perry, E., Ruffier, M., Tate, J., Thybert, D., Trevanion, S. J., Dyer, S., Harrison, P. W., Howe, K. L., Yates, A. D., Zerbino, D. R., and Flicek, P. (2022) Ensembl 2022. *Nucleic Acids Res* 50, D988-D995
17. Blum, M., Chang, H.-Y., Chuguransky, S., Grego, T., Kandasaamy, S., Mitchell, A., Nuka, G., Paysan-Lafosse, T., Qureshi, M., Raj, S., Richardson, L., Salazar, G. A., Williams, L., Bork, P., Bridge, A., Gough, J., Haft, D. H., Letunic, I., Marchler-Bauer, A., Mi, H., Natale, D. A., Necci, M., Orengo, C. A., Pandurangan, A. P., Rivoire, C., Sigrist, C. J. A., Sillitoe, I., Thanki, N., Thomas, P. D., Tosatto, S. C. E., Wu, C. H., Bateman, A., and Finn, R. D. (2021) The InterPro protein families and domains database: 20 years on. *Nucleic Acids Res* 49, D344-D354
18. Sigrist, C. J. A., de Castro, E., Cerutti, L., Cuche, B. A., Hulo, N., Bridge, A., Bougueleret, L., and Xenarios, I. (2013) New and continuing developments at PROSITE. *Nucleic Acids Res* 41, D344-347
19. Mistry, J., Chuguransky, S., Williams, L., Qureshi, M., Salazar, G. A., Sonnhammer, E. L. L., Tosatto, S. C. E., Paladin, L., Raj, S., Richardson, L. J., Finn, R. D., and Bateman, A. (2021) Pfam: The protein families database in 2021. *Nucleic Acids Res* 49, D412-D419
20. Durinck, S., Moreau, Y., Kasprzyk, A., Davis, S., De Moor, B., Brazma, A., and Huber, W. (2005) BioMart and Bioconductor: a powerful link between biological databases and microarray data analysis. *Bioinformatics* 21, 3439-3440
21. Durinck, S., Spellman, P. T., Birney, E., and Huber, W. (2009) Mapping identifiers for the integration of genomic datasets with the R/Bioconductor package biomaRt. *Nat Protoc* 4, 1184-1191
22. Medvar, B., Raghuram, V., Pisitkun, T., Sarkar, A., and Knepper, M. A. (2016) Comprehensive database of human E3 ubiquitin ligases: application to aquaporin-2 regulation. *Physiol Genomics* 48, 502-512
23. Szklarczyk, D., Gable, A. L., Lyon, D., Junge, A., Wyder, S., Huerta-Cepas, J., Simonovic, M., Doncheva, N. T., Morris, J. H., Bork, P., Jensen, L. J., and Mering, C. V. (2019) STRING v11: protein-protein association networks with increased coverage, supporting functional discovery in genome-wide experimental datasets. *Nucleic Acids Res* 47, D607-D613
24. Szklarczyk, D., Gable, A. L., Nastou, K. C., Lyon, D., Kirsch, R., Pyysalo, S., Doncheva, N. T., Legeay, M., Fang, T., Bork, P., Jensen, L. J., and von Mering, C. (2021) The STRING database in 2021: customizable protein-protein networks, and functional characterization of user-uploaded gene/measurement sets. *Nucleic Acids Res* 49, D605-D612
25. Henningsen, A., and Toomet, O. (2019) miscTools: Miscellaneous Tools and Utilities. 0.6-26 Ed., https://CRAN.R-project.org/package=miscTools
26. Wickham, H. (2016) ggplot2: Elegant Graphics for Data Analysis. *(Springer-Verlag New York)*
27. Wickham, H. (2007) Reshaping Data with the reshape Package. *Journal of Statistical Software* 21, 1-20
28. Charif, D., and Lobry, J. R. (2007) SeqinR 1.0-2: A Contributed Package to the R Project for Statistical Computing Devoted to Biological Sequences Retrieval and Analysis. In: Bastolla, U., Porto, M., Roman, H. E., and Vendruscolo, M., eds. *Structural Approaches to Sequence Evolution: Molecules, Networks, Populations*, pp. 207-232, Springer, Berlin, Heidelberg
29. Wickham, H. (2011) The Split-Apply-Combine Strategy for Data Analysis. *Journal of Statistical Software* 40, 1-29
30. Carlson, M. (2022) GO.db: A set of annotation maps describing the entire Gene Ontology. *https://doi.org/doi:10.18129/B9.bioc.GO.db*

31. Wickham, H. (2019) stringr: Simple, Consistent Wrappers for Common String Operations. https://CRAN.R-project.org/package=stringr
32. Slowikowski, K., Schep, A., Hughes, S., Dang, T. K., Lukauskas, S., Irisson, J.-O., Kamvar, Z. N., Ryan, T., Christophe, D., Hiroaki, Y., Gramme, P., Abdol, A. M., Barrett, M., Cannoodt, R., Krassowski, M., Chirico, M., and Aphalo, P. (2023) ggrepel: Automatically Position Non-Overlapping Text Labels with 'ggplot2'. https://CRAN.R-project.org/package=ggrepel
33. Kremer, L. P. M., and Anders, S. (2019) ggpointdensity: A Cross Between a 2D Density Plot and a Scatter Plot. https://CRAN.R-project.org/package=ggpointdensity
34. Chang, W. (2023) extrafont: Tools for Using Fonts. https://CRAN.R-project.org/package=extrafont
35. Wickham, H., Seidel, D., and Rstudio (2022) scales: Scale Functions for Visualization. https://CRAN.R-project.org/package=scales
36. Wagih, O. (2017) ggseqlogo: A 'ggplot2' Extension for Drawing Publication-Ready Sequence Logos. https://CRAN.R-project.org/package=ggseqlogo
37. Garnier, S., Ross, N., Rudis, B., Sciaini, M., Camargo, A. P., and Scherer, C. (2022) viridisLite: Colorblind-Friendly Color Maps (Lite Version). https://CRAN.R-project.org/package=viridisLite
38. Wickham, H., Hester, J., Ooms, J., Rstudio, Example, and Foundation, R. (2021) xml2: Parse XML. https://CRAN.R-project.org/package=xml2
39. Bache, S. M., and Wickham, H. (2022) magrittr: A Forward-Pipe Operator for R. https://CRAN.R-project.org/package=magrittr
40. Wickham, H., François, R., Henry, L., Müller, K., Vaughan, D., Software, P., and PBC (2023) dpylr: A Grammar of Data Manipulation. https://CRAN.R-project.org/package=dplyr
41. Wickham, H., Bryan, J., Posit, C., P. B., Kalicinski, M., Valery, K., Leitienne, C., Colbert, B., Hoerl, D., and Miller, E. (2023) readxl: Read Excel Files. https://CRAN.R-project.org/package=readxl
42. Briatte, F. (2023) ggnetwork: Geometries to Plot Networks with "ggplot2". https://cran.r-project.org/web/packages/ggnetwork/vignettes/ggnetwork.html
43. Csárdi, G., Nepusz, T., Traag, V., Horvát, S., Zanini, F., Noom, D., and Müller, K. (2024) igraph: Network Analysis and Visualisation in R. https://igraph.org/
44. Li, K., Vaudel, M., Zhang, B., Ren, Y., and Wen, B. (2019) PDV: an integrative proteomics data viewer. *Bioinformatics* 35, 1249-1251
45. Perez-Riverol, Y., Bai, J., Bandla, C., Garcia-Seisdedos, D., Hewapathirana, S., Kamatchinathan, S., Kundu, D. J., Prakash, A., Frericks-Zipper, A., Eisenacher, M., Walzer, M., Wang, S., Brazma, A., and Vizcaino, J. A. (2022) The PRIDE database resources in 2022: a hub for mass spectrometry-based proteomics evidences. *Nucleic Acids Res* 50, D543-D552
46. Chambers, M. C., Maclean, B., Burke, R., Amodei, D., Ruderman, D. L., Neumann, S., Gatto, L., Fischer, B., Pratt, B., Egertson, J., Hoff, K., Kessner, D., Tasman, N., Shulman, N., Frewen, B., Baker, T. A., Brusniak, M. Y., Paulse, C., Creasy, D., Flashner, L., Kani, K., Moulding, C., Seymour, S. L., Nuwaysir, L. M., Lefebvre, B., Kuhlmann, F., Roark, J., Rainer, P., Detlev, S., Hemenway, T., Huhmer, A., Langridge, J., Connolly, B., Chadick, T., Holly, K., Eckels, J., Deutsch, E. W., Moritz, R. L., Katz, J. E., Agus, D. B., MacCoss, M., Tabb, D. L., and Mallick, P. (2012) A cross-platform toolkit for mass spectrometry and proteomics. *Nat Biotechnol* 30, 918-920
47. MacLean, B., Tomazela, D. M., Shulman, N., Chambers, M., Finney, G. L., Frewen, B., Kern, R., Tabb, D. L., Liebler, D. C., and MacCoss, M. J. (2010) Skyline: an open source document editor for creating and analyzing targeted proteomics experiments. *Bioinformatics* 26, 966-968
48. Pino, L. K., Searle, B. C., Bollinger, J. G., Nunn, B., MacLean, B., and MacCoss, M. J. (2020) The Skyline ecosystem: Informatics for quantitative mass spectrometry proteomics. *Mass Spectrom Rev* 39, 229-244
